# How do substance and polysubstance use trajectories differ by sexual attraction from ages 17 to 24? A community-based longitudinal cohort study in Switzerland

**DOI:** 10.1136/bmjph-2025-003583

**Published:** 2026-01-27

**Authors:** Clarissa Janousch, Florian Vock, Babette L Winter, Tabea Hässler, Lukas Eggenberger, Laura Bechtiger, Michelle Loher, Tina Maria Binz, Markus R Baumgartner, Denis Ribeaud, Manuel Eisner, Boris B Quednow, Lilly Shanahan

**Affiliations:** 1 Department of Adult Psychiatry and Psychotherapy, University Hospital of Psychiatry Zurich, University of Zurich, Zürich, Switzerland; 2 Jacobs Center for Productive Youth Development, University of Zurich, Zürich, Switzerland; 3 Department of Global Public Health, Karolinska Institute, Stockholm, Sweden; 4 Swiss Aids Federation, Zürich, Switzerland; 5 Department Public and Global Health, University of Zurich Institute of Epidemiology Biostatistics and Prevention, Zürich, Switzerland; 6 Department of Psychology, University of Zurich, Zürich, Switzerland; 7 Population Research Center, University of Zurich, Zürich, Switzerland; 8 Department of Psychiatry, University Medical Centre Groningen, Groningen, Netherlands; 9 Zurich Institute of Forensic Medicine, University of Zurich, Zürich, Switzerland; 10 Institute of Criminology, University of Cambridge, Cambridge, UK

**Keywords:** Mental Health, Community Health, statistics and numerical data, Sexual Health, Drug Monitoring

## Abstract

**Introduction:**

Longitudinal, population-based studies of how youth substance use (SU) varies by sexual attraction, sex and their interaction remain scarce, especially in Europe. This study examines poly-SU (PSU) trajectories at 17, 20 and 24; disparities between sexual minority (SM) youth and heterosexual (HET) youth regarding these trajectories; and their correlates.

**Methods:**

We obtained data from the Zurich Project on Social Development from Childhood to Adulthood. SU was self-reported by n=1384 participants at 17, 20 and 24, and hair-tested at 20 and 24. Regression models included SM status, sex, their interaction, sociodemographic variables and psychosocial variables. This population-based longitudinal cohort analysis used linear mixed-effect models to examine developmental trajectories.

**Results:**

The proportion of SM youth increased from 11.3% at 17 to 23.4% at 24. At 20 and 24, SM were more likely than HET youth to have used cannabis, stimulants, ecstasy and hallucinogens in the past year. At 17, SM females reported high SU, which increased until 20, then plateaued or declined by 24, approaching those of HET males. SM males exhibited lower use at 17, but this sharply increased, reaching the highest levels by 24. HET females reported the lowest use. SM females had higher tobacco use, whereas SM males showed steep increases in alcohol, cannabis and stimulant use, surpassing all other groups regarding PSU by 24. Peer SU, low self-control, sensation-seeking and internalising symptoms predicted SU-related outcomes.

**Conclusions:**

Our findings on distinct developmental timing of the increases and escalation in SU highlight important opportunities for public health intervention, indicating that prevention efforts should be strategically timed and tailored to the unique escalation patterns observed among SM youth.

**WHAT IS ALREADY KNOWN ON THIS TOPIC**

- Sexual minority adolescents and young adults have higher rates of substance use and related harms, but longitudinal evidence on developmental trajectories from late adolescence into young adulthood in European population-based cohorts remains scarce.

**WHAT THIS STUDY ADDS**

- Using a community-based longitudinal cohort from Switzerland (ages 17–24), we show persistent disparities in single-substance and polysubstance use by sexual orientation and sex and examine associated psychosocial correlates.

**HOW THIS STUDY MIGHT AFFECT RESEARCH, PRACTICE OR POLICY**

- Findings support LGBTQ+-inclusive, developmentally timed prevention and harm-reduction strategies in late adolescence/young adulthood. They also underscore the importance of monitoring orientation-related disparities in routine surveillance.

## Introduction

 Most research on substance use (SU) among youth and young adults does not differentiate by sexual orientation. In this paper, we focus on sexual minority (SM) and heterosexual (HET) individuals across ages 17–24, capturing the transition from late adolescence (17 years) through emerging adulthood (20 years) into young adulthood (24 years). Ages 17–24 represent a key developmental window in which autonomy increases, peer contexts change and SU typically escalates and consolidates.^1^ Prior work in this cohort showed early emergence of SM-HET disparities but was limited to ages 17–20. We expand this evidence by adding age 24 data and modelling trajectories across the transition into young adulthood.^2^

Evidence indicates that SM individuals experience higher rates of SU and SU disorders than their HET counterparts (with studies typically including participants aged 18–30 years and rarely including participants younger than 18). This disparity represents a persistent public health concern. Research shows that SM adults are more than twice as likely to use illegal substances^3^ and develop SU disorders as compared with HET people.^4^ Such elevated rates have been documented across a range of substances, including tobacco,^5 6^ alcohol,^7 8^ cannabis,^9^ ecstasy/3,4-methylendioxymethylamphetamine (MDMA^10^), stimulants^11^ and opioids.^12^ However, some studies suggest that the difference in alcohol use can be less pronounced.^13^ Furthermore, although most research on the LGBTIQ+ population has focused on men who have sex with men,^14 15^ research indicates that SM women might be at a higher risk of elevated SU.^16^

The disparities in SU among SM and HET seem to emerge during adolescence and young adulthood.^17^ Two recent Swiss population-based studies found pronounced SU differences among female SM compared with female HET, especially for cannabis, whereas no such differences were observed among males.^2 18^ At age 17, female SM reported significantly higher rates of use for cannabis, ecstasy and some hallucinogens than female HET, and by age 20, female SM reported the elevated use of tobacco, stimulants and additional hallucinogens. In contrast, male SM youth reported lower rates of use for cannabis and tobacco as compared with male HET at age 17, but significantly higher rates of use for ecstasy and hallucinogens by age 20.^2^ The present study builds on these prior analyses by adding an assessment at age 24, allowing us to examine whether group differences persist over time or change as the participants transition into young adulthood.

In addition to higher rates of use for individual substances, poly-SU (PSU), use of multiple substances within the past 12 months, is more common among SM than HET. SM are more likely to combine tobacco, alcohol and cannabis, whereas HET typically use tobacco and alcohol only.^19^ This differs from simultaneous use, which emphasises concurrent consumption in a short window. Most existing studies are cross-sectional, limiting insight into SU trajectories across adolescence and young adulthood. Moreover, changes in reported sexual attraction over time (ie, sexual fluidity) are rarely considered in longitudinal SU research.^20^

### Sex differences within SM and HET

Rates of SU differ substantially by sex, and this further differs by SM status. Sex-based analyses indicate that female HET report lower SU rates than male HET.^21^ Studies in late adolescence and adult populations have found that female SM have comparable or even higher rates than male SM.^4 22^ Male SM are more likely to use cannabis, inhalants and stimulants compared with male HET, whereas female SM report the elevated use of cannabis and stimulants as compared with female HET.^3^ Sexualised SU, especially in chemsex (using substances to extend arousal),^23^ may influence use patterns in SM, though it has mainly been studied in adult male SM, with limited evidence available for adolescents or young SM individuals.^24^

### Explaining SU disparities: psychosocial theories

Three complementary frameworks help explain the disparities in SU between SM and HET. The Minority Stress Model^25 26^ posits that SM face chronic, identity-related stress due to stigma, discrimination and marginalisation. These stressors contribute to psychological distress and increase SM’s vulnerability to using substances as a coping strategy.^27^ This framework is often associated with the use of substances such as alcohol, tobacco or cannabis, which are commonly used for stress relief, emotional regulation or numbing. The Syndemic Theory^28^ builds on this model, suggesting that co-occurring problems, such as depression and SU, mutually reinforce one another, particularly in the context of overlapping structural vulnerabilities. Together, these frameworks emphasise that both external and internalised stress, coupled with social disadvantage, may increase the risk of SU and PSU among SM. Sociocultural and peer contexts can shape SU norms among youth,^29 30^ and some LGBTQ+ (lesbian, gay, bisexual, transgender, queer and more) environments may be more substance-tolerant, particularly for men.^31^ These influences, together with minority stress processes, may help explain elevated SU risk among SM youth. In addition, established psychosocial factors—including peer SU, sensation seeking, self-control, emotional distress and bullying—are consistently associated with adolescent SU^2 17 32^ and were therefore included as predictors.

### The present study

Existing studies, predominantly from the USA, show higher SU among SM youth, but offer limited guidance for European contexts. Longitudinal, population-based studies on SM-HET SU disparities in Europe are scarce, and most prior work is cross-sectional. Few studies combine repeated self-reports with biological markers such as hair toxicology to address reporting biases.^33^ This study is among the first in Switzerland to examine SU and PSU trajectories among SM youth using longitudinal data and comprehensive psychosocial predictors.

To address these gaps, we use longitudinal data obtained from a Swiss community-based cohort^34^ to investigate SU among HET and SM at ages 17, 20 and 24, the transition from late adolescence into young adulthood, allowing for examining SU disparities across this critical developmental period. In this study, we use the term ‘SM’ to refer to people reporting any level of same-sex attraction. Please note, however, that sexual attraction, behaviour and sexual orientation are correlated but distinct constructs.^35 36^ SU was assessed through self-reports at all three time points, with additional hair test data available at ages 20 and 24 to complement the survey responses.

The study had four main objectives. First, we examined the development of sexual attraction from ages 17 to 24. Rather than assuming changes in reported attraction reflect actual sexual fluidity, we conceptualise such shifts as indicative of developmental processes of identity disclosure (‘coming out’), consistent with prior literature showing that awareness of sexual orientation precedes self-identification,^37^,^39^ making it likely that classification changes from HET to SM. Additionally, sexuality can be fluid over time, and sexual diversity develops through dynamic processes rather than being rigid or categorical.^40^ We therefore expect that some participants may report changes in their attraction across assessment waves and account for these reported changes at each time point (To evaluate whether time-varying sexual orientation may bias results, we further conducted additional sensitivity analyses using baseline-only (age 17) and time-invariant (‘ever SM’) operationalisations of sexual orientation. These analyses supported the robustness of the main findings.). Second, we compared SM and HET youth regarding psychosocial stressors, expecting that SM would report higher levels, due to experiences of discrimination and marginalisation.^26 41^ Third, we investigated substance-specific SU and PSU trajectories across SM status, sex and their interaction, anticipating consistently higher levels among SM and potential interaction effects.^2 19 22^ Fourth, we examined whether psychosocial stressors predict SU trajectories and help explain observed group disparities, expecting that stressors particularly relevant to SM would significantly predict elevated SU.^2^ Finally, as an exploratory step, we compared self-reported SU data with objective hair data (ages 20 and 24) to assess concordance and support the validity of participants’ reported SU trajectories, though hair data were not used in inferential models due to sample size limitations.^31 42^

## Materials and methods

### Recruitment and participants

Participants were drawn from the Zurich Project on Social Development from Childhood to Adulthood (z-proso^34^), an ongoing longitudinal cohort study initiated in 2004. The original target sample comprised 1675 participants from 56 primary schools in the city of Zurich, selected through stratified random sampling to ensure representation across various socioeconomic backgrounds. Across all assessment waves, data were obtained from 1583 participants (94.5% of the initial sample), each contributing information at least once at nine main data waves, either directly or through another informant. The current analysis focuses on SU data collected at waves 7–9, corresponding to ages 17, 20 and 24, with a few additional constructs measured at earlier waves. We refer to the three assessment ages as late adolescence (17 years), emerging adulthood (20 years) and young adulthood (24 years). Participants were included if they provided at least one valid data point on sexual attraction and SU across these waves (n=1384). Sample retention was high (95.6% at 17, 88.1% at 20, 84.2% at 24, see online supplemental table S1). All data used in this study were pseudonymised before analysis and stored separately from identifying information, which is not accessible to users of the data. Data, including information on sexual attraction, were collected through secure electronic questionnaires, and confidentiality was ensured according to Swiss data protection standards. As the study uses fully pseudonymised cohort data and contains no identifiable individuals or case details, patient consent for publication was not required.

### Measures

Please refer to online supplement for detailed or additional information on all measures.

Past-year SU frequency for tobacco, alcohol, cannabis, ecstasy/MDMA, stimulants such as cocaine and (meth-)amphetamine, hallucinogens, benzodiazepines and opioids was assessed at ages 17, 20 and 24 using standard 6-point frequency scales (details in online supplement).^43^ For the hair data, participants provided samples at ages 20 and 24, as objective indicators of SU. Analytical procedures and detection thresholds are reported in online supplement.^44 45^ PSU variables were operationalised using three indices (Poly1–3) reflecting different substance groupings across waves (details in online supplement).^32^ Sexual attraction was self-reported at each wave, using a 5-point scale. Individuals indicating any same-sex attraction were classified as SM, others were classified as HET (details in online supplement). Sociodemographic variables included sex assigned at birth, parental migration background, family socioeconomic status (SES) (international socio-economic index (ISEI)^46^) and educational level.^32^ Coding details and distributions are provided in online supplement. Psychosocial and behavioural variables were selected based on their established associations with adolescent and young adult SU and related problem behaviours,^17 32^ and to align with previous analyses conducted with the same cohort.^2^ Peer SU,^47^ sensation-seeking,^48 49^ low self-control,^50^ internalising symptoms,^51^ bullying victimisation^52^ and leisure activities^47^ were assessed using validated scales. Information on items, reliability coefficients, coding and wave-specific availability is detailed in online supplement.

### Statistical analysis

**Figure 1:**
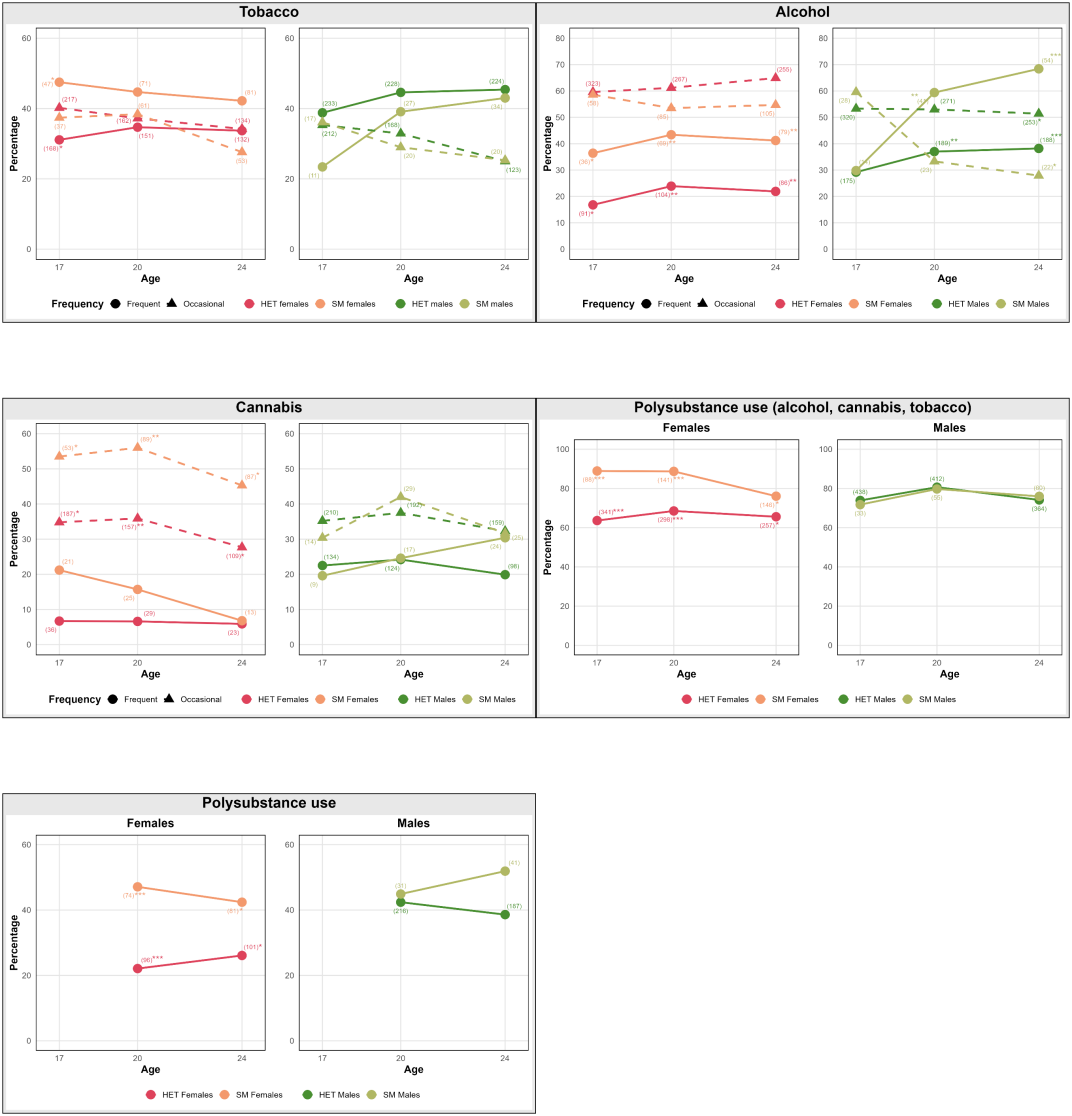
Trajectories of tobacco, alcohol, cannabis, polysubstance use (alcohol, cannabis, tobacco; poly1) and polysubstance use (poly3) with at least one significant difference between heterosexual (HET) and sexual minority (SM) youth and young adults from ages 17 to 24. Note: asterisks indicate significance. *p<0.05, **p<0.01, ***p<0.001. Sample sizes in brackets. The last graph shows poly 3 (≥2 of 12 substances excluding alcohol, tobacco and cannabis). All other substances and polysubstance use patterns not displayed showed no significant differences.

**Figure 2:**
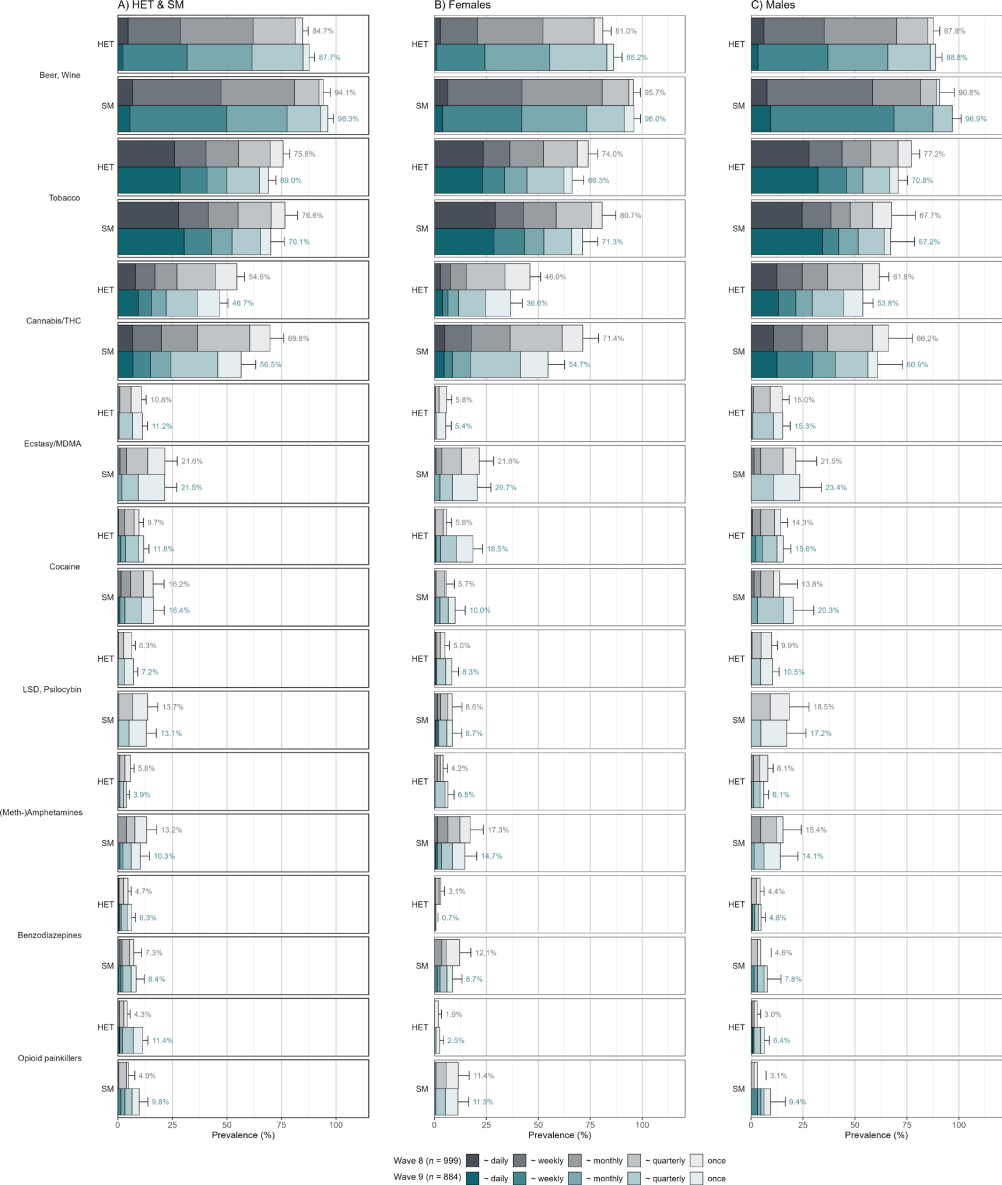
Self-reported 12-month prevalence rates at ages 20 and 24 with SM/HET groupings at each time point separately. Note: Wave 8 equals age 20, Wave 9 equals age 24. HET, heterosexual; LSD, lysergic acid diethylamide; MDMA, 3,4-methylendioxymethylamphetamine; SM, sexual minority; THC, Δ9-tetrahydrocannabinol.

We conducted descriptive and longitudinal analyses of SU trajectories at ages 17, 20 and 24. Trajectory graphs illustrated changes in prevalence over time, and z-tests for proportions tested group differences at each time point (*p<0.05, **p<0.01, ***p<0.001). High-prevalence substances (tobacco, alcohol and cannabis) were categorised into frequent (ie, weekly or (almost) daily use) and occasional use (ie, less than weekly use) to provide a nuanced depiction of use patterns. Any other substance (ie, ecstasy, (meth-)amphetamines, cocaine, stimulants, hallucinogens, opioids and benzodiazepines) was coded as a binary (use vs no use) due to lower prevalence (figure 1; online supplemental figures 1 and 2a–c). To model developmental trajectories, we used linear mixed-effects models with repeated measures nested within individuals. Missing data were not imputed. However, linear mixed-effects models were estimated using restricted maximum likelihood, allowing the inclusion of all available observations per participant. Age was entered as a continuous variable (linearly and quadratically) to capture curvilinear patterns.^53^ Substances assessed at only two waves (benzodiazepines, opioids, poly 3) were analysed linearly. No formal corrections for multiple testing were applied, as analyses were exploratory. Effect sizes and estimates (β, SE and 95% CI where applicable) were reported consistently. Interaction effects (sex×sexual attraction) were tested for each substance and PSU category. Models were built stepwise: (1) baseline (age, sexual attraction, sex), (2) sociodemographic (adding migration background, ISEI, education) and (3) full model (adding psychosocial/behavioural predictors described above). Interaction effects (sex×sexual attraction) were interpreted using model coefficients and visualised via predicted trajectories. No formal contrast testing was conducted, as analyses were exploratory and primarily descriptive. Hair toxicology data were not included in statistical models, due to limited availability and convergence issues; however, they were analysed descriptively to validate self-reported SU patterns across groups (see figures2  3). Analyses were conducted in R V.4.2.2^54^ using psych,^55^ tidyverse,^56^ rstatix,^57^ ggplot2,^58^ lme4^59^ and lmerTest.^60^

**Figure 3:**
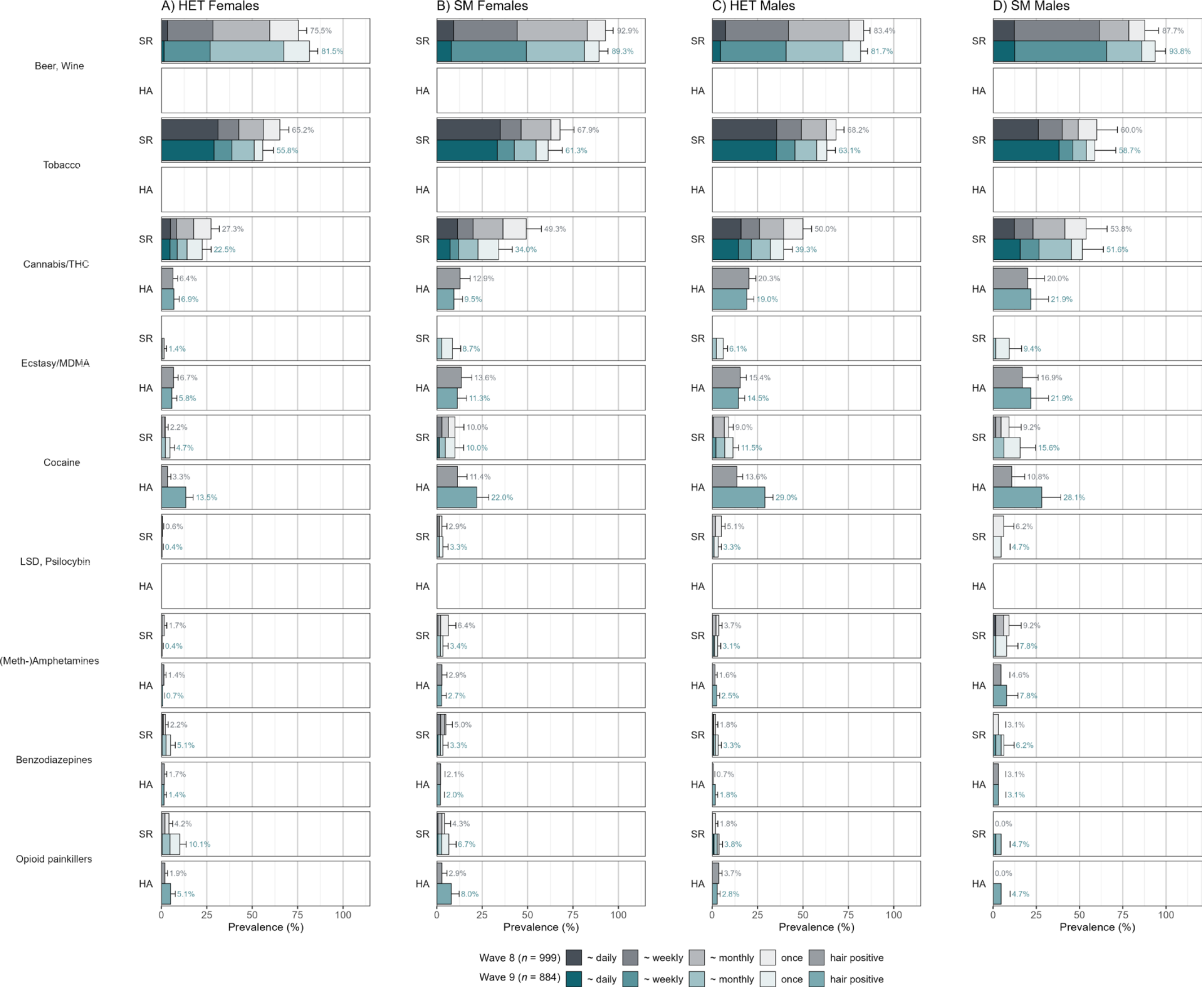
3-month hair and self-reported prevalence rates at ages 20 and 24 with SM/HET groupings at each time point separately. Note. Wave 8 equals age 20, Wave 9 equals age 24. Empty cells indicate that no data were available for that substance/time point. HA, hair-analysed; HET, heterosexual; LSD, lysergic acid diethylamide; MDMA, 3,4-methylendioxymethylamphetamine; SM, sexual minority; SR, self-report; THC, Δ9-tetrahydrocannabinol.

### Patient and public involvement

This research was conducted without patient or public involvement and was not pre-registered. We followed the Strengthening the Reporting of Observational Studies in Epidemiology statement for the reporting of observational studies.^61^

## Results

### Sample characteristics and sexual attraction changes

**Figure 4:**
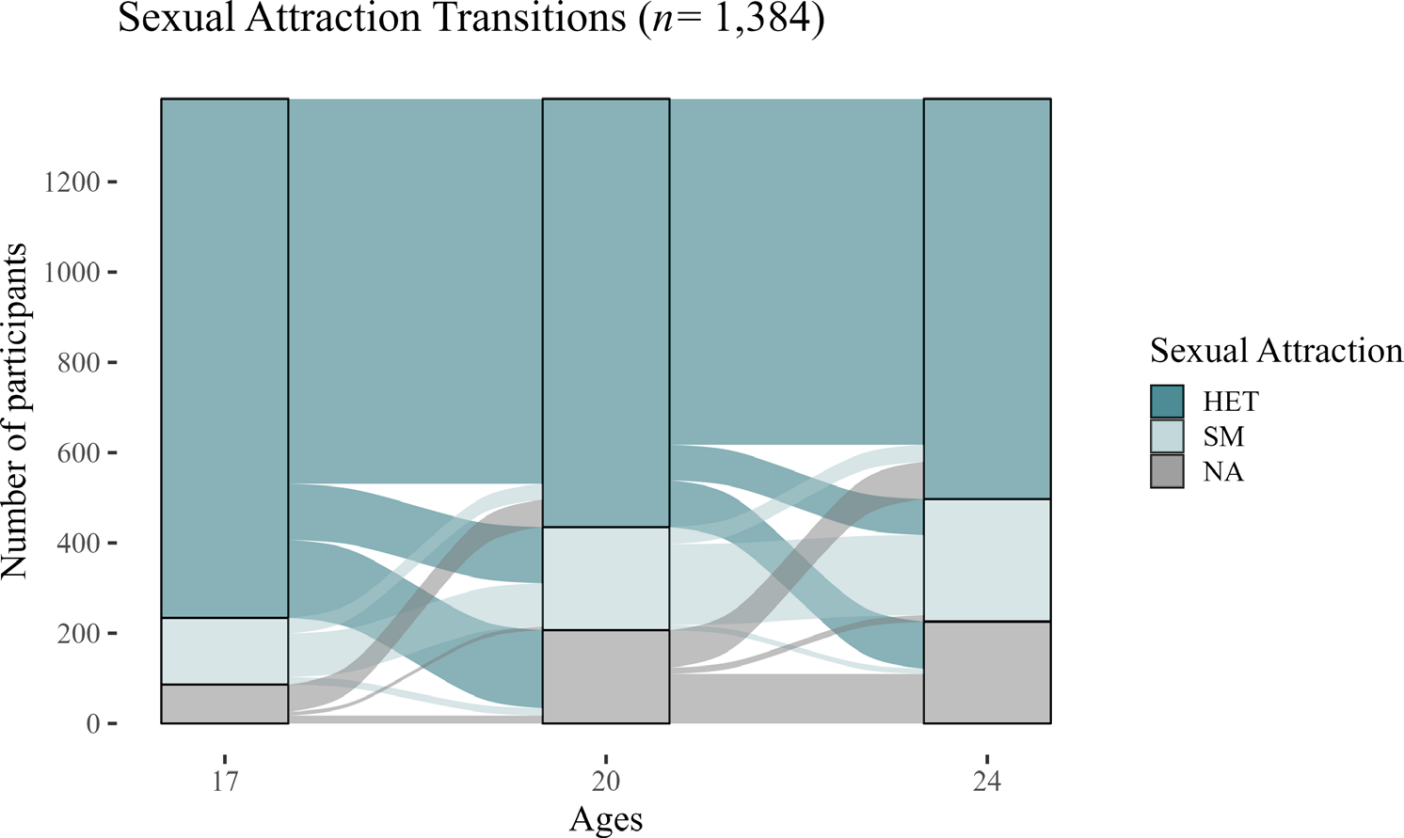
Changes in sexual attraction between the ages of 17 and 24. HET, heterosexual; NA, not available/no data; SM, sexual minority.

Between ages 17 and 24, the prevalence of SM increased from 11.3% at age 17 (n=147/1297; females 15.5%, males 7.2%) to 23.4% at age 24 (n=271/1158), indicating sexual attraction fluidity (figure 4, online supplemental table S1). Women consistently reported higher rates of same-sex attraction compared with men across all waves (age 24: females 32.8%, males 13.8%).

Group differences in sociodemographic and psychosocial characteristics are summarised in online supplemental table S1. Although raw group comparisons showed only small variations in SES, education and migration background, regression models revealed more consistent patterns, particularly for migration background. Psychosocially, SM youth reported higher internalising symptoms, more bullying victimisation (especially among males at age 17), and greater exposure to substance-using peers compared with HET youth, particularly at age 20. Other variables, such as sensation-seeking, self-control and engagement in leisure activities, showed minor or inconsistent differences across groups.

### Longitudinal trajectories of SU

Trajectories of self-reported SU differed notably between SM and HET (figure 1, online supplemental S1) and between females and males (online supplemental figure S2a–c). SM youth, particularly SM females during late adolescence and SM males in early adulthood, consistently reported higher levels of use compared with their HET peers. While SM females reported elevated SU as early as age 17, with patterns stabilising or slightly declining by age 24, SM males exhibited comparatively lower use at 17 but showed a marked increase between ages 20 and 24, ultimately surpassing all other groups by young adulthood. In contrast, HET females consistently reported the lowest levels across substances and time. Frequent use increased more sharply over time than occasional use, particularly among males. SM females maintained the highest levels of tobacco use from adolescence onward, whereas SM males showed a strong increase in alcohol, cannabis and stimulant use across time. For cannabis, female SM youth initially reported the highest occasional use, but their levels declined by age 24, whereas SM males became increasingly frequent users over time. Similar trajectories were observed for ecstasy/MDMA and other stimulant substances, with SM males showing the steepest increase by age 24. For benzodiazepine and opioid use, sex differences were more pronounced than SM status differences, with females reporting higher levels than males overall. Regarding PSU, SM females showed the highest levels of combined tobacco, alcohol and cannabis use (Poly1), whereas SM males had the steepest increase in broader PSU categories (Poly2 and Poly3), reaching the highest levels by age 24.

### Predictors of SU and PSU

**Figure 5:**
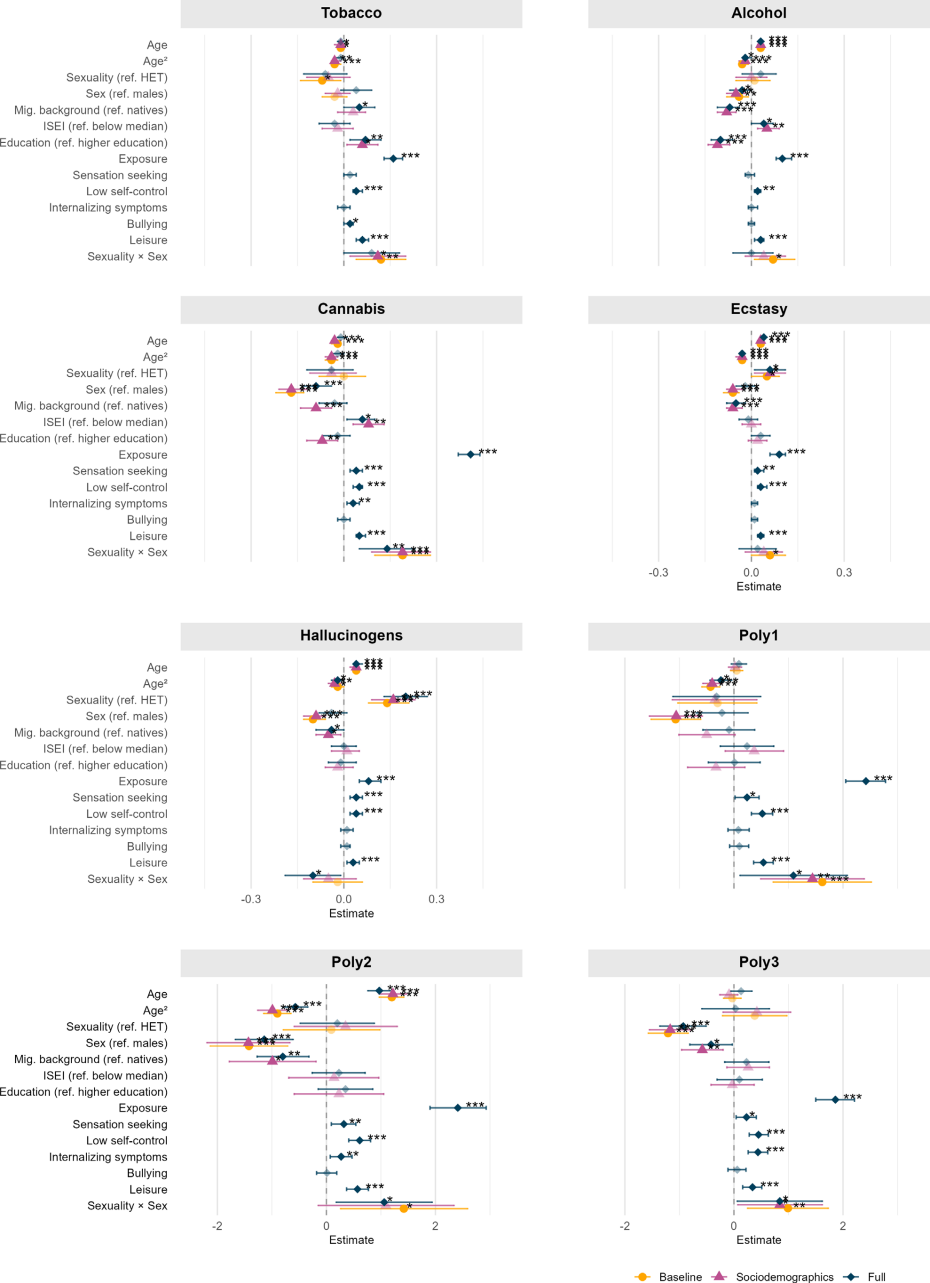
Predictors of substance and polysubstance use outcomes with significant sex×sexuality interaction effects in at least one of the three models (baseline, baseline+demographics, baseline+demographics+psychosocial). HET, heterosexual; ISEI, international socio-economic index.

Baseline regression analyses confirmed significant differences between SM and HET youth across multiple substances, including alcohol, cannabis, ecstasy/MDMA, stimulants, cocaine, (meth-)amphetamines, hallucinogens and PSU categories (online supplemental table S2a). Interaction terms between sex and sexual attraction at baseline indicated significantly higher vulnerability among SM females compared with males. Specifically, significant sex×sexual attraction interactions were observed for tobacco, alcohol, cannabis, ecstasy/MDMA and all PSU categories. These interactions consistently highlighted elevated SU, specifically among SM females relative to males (figure 5, online supplemental table S2b).

After adjusting for sociodemographic covariates (including parental migration background, SES and educational background), SM status remained a robust predictor of elevated SU for most substances. Socioeconomic indicators demonstrated variable significance: parental migration background generally predicted lower SU, whereas higher educational attainment was associated with differentially elevated SU depending on the specific substance (online supplemental table S3a). In the sociodemographic-adjusted models, significant interaction terms between sex and sexual attraction persisted for several substances, confirming that SM females remain particularly vulnerable even after accounting for socioeconomic background. Notably, significant sex×sexual attraction interactions were identified for tobacco and cannabis, and the polysubstance categories, Poly 1 and Poly 3 (figure 5, online supplemental table S3b).

When including all sociodemographic and psychosocial covariates, SM status continued to be a significant predictor of increased SU across most substances and polysubstance categories. Psychosocial factors also showed strong, consistent associations. Higher sensation-seeking scores significantly predicted increased SU across most substances and polysubstance categories. Elevated internalising symptoms consistently emerged as significant associations, emphasising the role of emotional distress in SU among adolescents and young adults. Additionally, the influence of peers on SU-related behaviours was a robust and significant predictor across nearly all substances, further underscoring the influence of peers on substance-related behaviours (online supplemental table S4a). In the fully adjusted models, significant interactions between sex and sexual attraction persisted, most notably for cannabis, hallucinogens and all PSU categories (figure 5, online supplemental table S4b). An overview of significant associations for the final interaction model is provided in online supplemental table S4c.

### Prevalence of self-reported and hair-assessed SU at ages 20 and 24

In addition to the longitudinal trajectories, we examined self-reported and hair-assessed SU at ages 20 and 24. These analyses provided a more detailed picture of SU patterns, including substances not assessed at age 17, and allowed for cross-validation of self-reported data through hair testing. However, further statistical analyses of predictors were not stable due to the limited availability of hair data at only two time points.

The 12-month self-reported prevalence rates revealed significant disparities between SM and HET youth at ages 20 and 24. SM consistently reported higher prevalence rates across most substances, notably THC (Δ9-tetrahydrocannabinol, the primary psychoactive component of cannabis measured in hair), ecstasy/MDMA, stimulants (including cocaine and (meth-)amphetamines) and hallucinogens (figure 2).

Notably, THC can only be reliably detected in hair from individuals with frequent or heavy cannabis use, limiting its sensitivity for occasional users.^62 63^ Disparities between SM and HET were pronounced for stimulants and MDMA, substances typically associated with recreational nightlife contexts. In hair analysis, disparities in the 3-month prevalence of substances were further accentuated, especially for substances associated with greater social stigma. SM and HET youth displayed higher hair-analysed prevalence rates for stimulants (eg, cocaine, amphetamines) and prescription opioids compared with self-reported data, suggesting systematic underreporting biases among SM in self-reported measures (figure 3), as seen in previous studies of youth.^42 62^

## Discussion

This study tracked SU and PSU trajectories from ages 17 to 24 among SM and HET youth in a large urban Swiss cohort. SM youth, particularly females in adolescence and males in early adulthood, consistently reported higher SU than HET peers, with disparities widening over time. Frequent use of tobacco, alcohol, cannabis and party drugs (eg, ecstasy/MDMA, stimulants) increased notably among SM youth, and PSU was most prevalent among SM females, especially for combined alcohol, cannabis and tobacco use. These findings highlight the developmental nature of SU disparities and underscore critical timing windows for targeted prevention, earlier for SM females and later for SM males.

### Developmental timing and trajectory differences

The distinct developmental trajectories suggest that SU disparities among SM evolve dynamically across adolescence and early adulthood. In female SM, elevated SU risk was already evident by age 17 and remained high, whereas male SM showed a delayed but sharp increase in SU, particularly between ages 20 and 24. These diverging patterns may reflect differences in identity development timelines and community integration. For instance, gendered social norms may shape how and when youth disclose or act on same-sex attraction. Among male SM, stronger stigma and social pressure may contribute to underreporting or later outward expression, potentially delaying engagement with LGBTQ+ communities. In contrast, SM females may differentiate earlier from HET peers, leading to earlier SU increases, similar to HET males. By ages 20 and 24, participation in LGBTQ+ nightlife and peer networks may further amplify SU, particularly among male SM in urban settings like Zurich. Although some studies suggest similar or even earlier identity development among SM males (eg,^64^ the timing and social context of disclosure and community engagement likely vary by gender, setting, culture and geographic location).

### Psychosocial mechanisms

Findings indicating elevated SU among SM youth align with the Minority Stress theory,^25 26^ which posits that chronic stigma increases psychological distress and vulnerability to SU. Indeed, SM in this study reported higher levels of internalising symptoms and bullying victimisation compared with HET youth, which may link minority status to SU. The results also resonate with the Syndemic Theory,^28^ particularly the co-occurrence of psychosocial vulnerabilities and elevated PSU among SM. This suggests a cumulative risk process in which co-occurring adversities interact to amplify health risks.

### Sociocultural environment

At the same time, our findings extend beyond stress-based models. Elevated stimulant, ecstasy/MDMA and PSU among SM, particularly females, support emerging sociocultural perspectives that conceptualise SU as embedded in community norms, nightlife practices and identity expression.^65^ In urban contexts, such as Zurich, SU is often embedded in recreational and social settings. Queer-coded spaces, particularly those tied to electronic music and techno culture, with their longstanding association with SM communities, may represent settings where substances are more tolerated or accessible for some individuals, although practices vary widely across LGBTQ+ spaces. This aligns with research indicating that social and cultural environments, including peer norms, nightlife participation and community-based identity expression, can influence SU among SM populations. Large-scale events such as the Street Parade and Zurich Pride reflect the visibility and diversity of these intersecting scenes. For some, SU may serve as a coping mechanism as well as a means of social integration or enhancing intimacy, consistent with observations of sexualised SU and chemsex practices, particularly among men having sex with men.^66^

Furthermore, the political context needs to be taken into account. In Switzerland, SMs still face significant prejudice and discrimination.^38^ However, SM is generally widely accepted and protected by Swiss law. In contrast, more than 60 countries criminalise SM,^67^ which may increase risky SU among SM populations. Additionally, legal access to certain substances during adolescence and early adulthood may also influence developmental trends in SU. Alcoholic beverages with <16% alcohol (eg, beer and wine) and tobacco products are legally purchasable from age 16, whereas spirits are available from age 18 onwards. Previous research has shown that a substantial proportion of adolescents tries substances a year before they can legally buy them.^43^ Cannabis remains illegal for recreational use (<1% THC), although public debate and tolerance practices are ongoing.^68^ These regulatory conditions shape availability and social acceptance, particularly from age 16 onward, and should be considered when interpreting age-related increases in SU.

This framework also contextualises our PSU findings. High PSU among SM females highlights a high-risk profile not fully captured by single-substance models.^32^ These patterns reinforce the need for public health approaches that recognise both the individual vulnerabilities and respect the sociocultural contexts that shape SU behaviour among SM. Furthermore, the high SU observed among female SM indicates that services targeting queer females (and non-binary youth) are necessary, and that studies on LGBTIQ+ populations should become more inclusive of understudied populations within the community.^14^

Finally, hair toxicology data at ages 20 and 24 among a subsample of participants provided a valuable lens for validating self-reported SU patterns. The objective SU data largely confirmed trends observed in self-reports, but also revealed higher prevalence rates for substances typically underreported due to stigma, such as opioids and stimulants.^42^ Although the sample size for hair data was smaller, it offered crucial methodological triangulation and supports future integration of objective measures in SU research.

### Strengths and limitations

Several key strengths can be addressed. First, this study draws on longitudinal data spanning from late adolescence to early adulthood, a period during which SU patterns unfold. The use of these three assessment waves for most self-reported measures allowed for detailed trajectory modelling and subgroup comparisons based on sexual attraction and sex. Second, the study included a large urban community sample assessing SU among both HET and SM, enabling robust analyses of the effect of SM status, sex and their intersection, which are rarely feasible in general population cohorts. Third, self-reported SU was complemented with objective hair toxicology among a subset of participants, enhancing the validity of findings and addressing concerns about potential reporting biases. Finally, including multiple psychosocial predictors allowed for nuanced exploration of underlying risk mechanisms.

However, the study also has several limitations. First, SM status was assessed via sexual attraction only, without additional indicators such as sexual identity or behaviour. Because attraction, behaviour and identity are distinct constructs, this underrepresents the complexity of SM status. Moreover, the study did not directly ask participants about their sexual orientation, which may have excluded or misclassified individuals with identities (eg, asexual, or those primarily attracted to non-binary or transgender people). We further did not distinguish between mono- and plurisexual SM. Yet, Swiss data reveal that plurisexual individuals face more discrimination and receive less support, both from inside and outside the LGBTIQ+ community.^69^ Furthermore, changes in reported attractions across waves may reflect identity disclosure, since the coming out process often occurs during this age range,^38^ potentially leading to some misclassification. Second, the male SM subgroup was relatively small, particularly in early waves, which may limit statistical power. Underreporting of SM identification due to social stigma is quite common.^70^ Given that societal norms towards male same-sex attraction are more negative than compared with female same-sex attraction,^71^ underreporting might have been more pronounced among male participants. Third, the present study assessed sex in a binary fashion, without distinguishing between sex and gender. We further did not assess transgender and intersex status. As a result, gender-diverse and intersex individuals may be underrepresented or misclassified. Future studies should go beyond the sex and gender binary and also assess people’s transgender status, aiming to be more inclusive of people who fall outside of the sex and/or gender spectrum.^14 72 73^ Fourth, the legal and social acceptance of SM varies dramatically across countries, with more than 60 nations still criminalising SM people.^67^ However, the vast majority of research is conducted in relatively tolerant Western countries.^14^ To better understand how context influences the experiences of SM people, future research should intensify efforts to collect data from Africa, Asia and Central America, while prioritising the safety of the involved scholars and participants. Fifth, hair toxicology data were only available at ages 20 and 24, and not all participants provided samples, limiting representativeness. Women and those with a migration background were less likely to participate (see ^74^ for details). Additionally, due to the limited number of valid hair samples, particularly among SM males, models using hair data as outcomes did not converge for less common substances, and hair concentrations were therefore used only to support the validity of self-reported SU. Sixth, SU is always highly context-specific. This study focuses on youth in an urban Swiss context with specific cultural and structural characteristics, limiting its generalisability particularly to rural settings, non-European contexts, or populations with differing legal, cultural or structural conditions related to SU and sexuality. Finally, although psychosocial factors were included, minority stress-specific variables were not explicitly measured, which limits the ability to formally test theoretical frameworks.

### Implications for prevention, intervention and future research

Elevated SU emerges by age 17 among female SMs and rises sharply among male SM in early adulthood, highlighting the need for targeted, developmentally sensitive prevention and intervention strategies. Efforts should focus on school-based programmes during critical transition periods and span multiple settings: schools, youth services, primary care, and, where appropriate, LGBTQ+ community spaces or nightlife-based harm-reduction initiatives address not only SU but also underlying psychosocial factors, including emotional distress and peer influence. Inclusive, identity-sensitive approaches are essential. Because SM youth often anticipate rejection,^75^ educators, parents and peers should actively communicate acceptance and support, fostering safer, more affirming environments that reduce vulnerability and promote healthy development.

Future research should aim to more directly assess the structural and cultural mechanisms hypothesised to underlie SU disparities, ideally in large, representative cohort studies and possibly include earlier timepoints.^13^ Cross-cultural comparisons could help understand whether similar patterns arise in different policy or social environments and assess mechanisms more directly, such as minority stress pathways or access to community resources. In addition, studies should move beyond damage-centred frameworks by also highlighting protective and community-based factors, such as resilience, social support and inclusive environments.^76^ Stronger intersectional designs are needed to better capture diversity within sexual and gender minority groups, as studies have shown differences, for example, between lesbian and bisexual women regarding their SU^77^ as well as among transgender and gender-diverse individuals, who may have a range of sexual orientations.^78^

## Conclusion

This study provides a longitudinal investigation of SU disparities between SM and HET youth in a European context, tracking developmental trajectories from adolescence into early adulthood. The findings reveal persistent and, in some cases, widening disparities in SU and PSU between SM and HET, particularly among females. These disparities were not fully explained by established psychosocial risk factors, underscoring the importance of considering minority-specific stressors and sociocultural influences. This study highlights the need for timely, tailored, culturally and contextually sensitive prevention efforts by integrating objective biomarkers and examining subgroup-specific patterns over time. These findings provide actionable evidence for tailoring early intervention and harm-reduction strategies to SM youth, with attention to sex-specific developmental timing and integration across school, healthcare and community settings.

## Supplementary material

10.1136/bmjph-2025-003583 Supplementary file 1

## Data Availability

Data are available upon reasonable request.
