# Supplementary material for: How do substance and polysubstance use trajectories differ by sexual attraction from ages 17 to 24? A community-based longitudinal cohort study in Switzerland
Source: BMJ Public Health. 2026 Jan 27;4(1):e003583. doi: 10.1136/bmjph-2025-003583 (PMC12853546; doi:10.1136/bmjph-2025-003583)
Supplement: Supplementary file 1 [file bmjph-4-1-s001.pdf]

How do substance and polysubstance use trajectories differ by sexual attraction from ages 17–24? A community-based longitudinal cohort study in Switzerland

SUPPLEMENT

Clarissa Janousch, Florian Vock, Babette L. Winter, Tabea Hässler, Lukas Eggenberger, Laura Bechtiger, Michelle Loher, Tina M. Binz, Markus R. Baumgartner, Denis Ribeaud, Manuel Eisner, Boris B. Quednow, Lilly Shanahan

Measures. Detailed information on measures. 2

References for Measures 4

Table S1. Descriptives. 6

Table S2a. Baseline models without interaction terms 7

Table S2b. Baseline models with interaction terms....................9

Table S3a. Sociodemographic models without interaction terms 12

Table S3b. Sociodemographic models with interaction terms 15

Table S4a. Full models without interaction terms....................18

Table S4b. Full models with interaction terms 23

Table S4c. Overview of significant predictors. Full models (baseline + demographics + control) with interaction terms....................30

Figure S1. Trajectories of Ecstasy, cocaine, (meth-)amphetamine, stimulant, hallucinogen, opioid, benzodiazepine, and poly2 use with no significant difference between heterosexual (HET) and sexual minority youth and young adults (SM) from ages 17 to 24. 31

Figure S2a. Trajectories of tobacco, alcohol, and cannabis use among male and female youth and young adults, stratified by sexual attraction (HET vs. SM) from ages 17 to 24. 32

Figure S2b. Trajectories of Ecstasy, cocaine, (meth-)amphetamine, stimulant, hallucinogen, opioid, and benzodiazepine use among male and female youth and young adults, stratified by sexual attraction (HET vs. SM) from ages 17 to 24. 33

Figure S2c. Trajectories of polysubstance use (Poly1-3) among male and female youth and young adults, stratified by sexual attraction (HET vs. SM) from ages 17 to 24. 34

Measures. Detailed information on measures.

SU was assessed through self-reported questionnaires at ages 17, 20, and 24, and hair at ages 20 and 24. Participants reported their use of various substances, including tobacco, alcohol, cannabis, Ecstasy/MDMA, stimulants such as cocaine and (meth-)amphetamine, hallucinogens, benzodiazepines, and opioids [1]. Additionally, we used a combined stimulant variable, aligned with previous work, including cocaine and (meth-)amphetamines [2]. Participants rated their 12-months SU frequency using a six-point scale (1 = *never*; 2 = *once*, 3 = *2–5 times*; 4 = *6–12 times [monthly]*; 5 = *13–52 [weekly]*; 6 = *53–365 times [daily]*). For the hair data, substances and their metabolites [pg/mg, log-transformed] were analyzed using liquid chromatography-tandem mass spectrometry. Details of this method have been described previously in other studies using z-proso data [3], [4].

PSU variables were created using self-reports. PSU refers to the use of two or more substances within the past 12 months, without inferring concurrent or closely timed use in this study. Three different versions of the PSU were coded, based on the availability of longitudinal data and Steinhoff et al. [5].

- Poly1 (ages 17, 20, 24): PSU of alcohol, tobacco, and cannabis (≥2 of 3 substances).
- Poly2 (ages 17, 20, 24): PSU of cannabis, Ecstasy/MDMA, cocaine, (meth-)amphetamines, and LSD/psilocybin. ≥2 of 5 substances excluding alcohol, tobacco, and cannabis.
- Poly3 (ages 20, 24): PSU of cannabis, cannabidiol, synthetic cannabis, Ecstasy/MDMA, (meth-)amphetamines, cocaine, LSD/psilocybin, 2C-drugs, ketamine, codeine, opioids, and benzodiazepines. ≥2 of 12 substances excluding alcohol, tobacco, and cannabis.

For each PSU variable, we summed the number of substances and coded each participant as PSU=1 if the sum was ≥2, and PSU=0 otherwise.

Sexual attraction

SM status was determined based on self-reported sexual attraction at each time point, beginning at age 17. Participants were classified as SM if they reported any level of same-sex attraction, on a scale from 1 = *I am attracted only to men* to 5 = *I am attracted only to women*; otherwise, they were categorized as heterosexuals (HET).

Sociodemographic variables:

Sex assigned at birth was measured as a binary variable (0 = *male*, 1 = *female*). Parental migration background was measured as a binary variable (0 = *at least one parent born in Switzerland*; 1 = *both parents born abroad*). Family Socioeconomic Status (ISEI) was assessed at ages 13/15 using the International Socio-Economic Index of Occupational Status (ISEI) based on parental occupation, ranging from 16 (e.g., unskilled worker) to 90 (e.g., judge; [6]). Education level was coded as a binary variable (0 = *higher*; 1 = *lower level*), reflecting participants' highest level of completed education at each time point, as in previous studies [5].

Psychosocial and behavioral variables:

The following variables were selected based on their established associations with adolescent and young adult SU and related problem behaviors [5], [7], and to align with previous analyses conducted with the same cohort [2]. In contrast to the previous analysis by Vock et al. (2023), subjective stress was excluded from the current models due to high multicollinearity with internalizing symptoms and because it was only assessed at ages 20 and 24.

Peer SU: Participants were asked about the influence of their two best friends and their intimate partner's illegal SU on a binary scale [8] at ages 17, 20, and 24. The final binary composite score indicated whether at least one of the three people indicated had used illegal substances in the past 12 months.

Sensation-seeking: Sensation-seeking was assessed at age 7 using an adapted nine-item version of the Travel Game developed by Alsaker and Gutzwiller-Helfenfinger [9], as described in Murray et al. [10]. The game used illustrated cardboard cards to present children with a series of choices between sensational and non-sensational travel scenarios (e.g., "Would you rather travel by fast motorbike or funny steam locomotive?"), with each sensational option scored as 1 and each non-sensational option as 0. A total sum score was calculated and rescaled to range from 0 to 1, with higher scores indicating greater sensation-seeking tendencies at age 7.

Low Self-control: Self-control was measured using a ten-item scale that covered five dimensions: risk-seeking, impulsivity, self-centeredness, preference for physical activity, and short-temperedness, adapted from Grasmick et al. 1993 [11]. Participants rated the items on a four-point Likert scale, ranging from 1 = *fully untrue* to 4 = *fully true*. Cronbach's alpha ranged between .73 and .74 at ages 17, 20, and 24.

Internalizing symptoms: Nine items from the Social Behavior Questionnaire were used to measure internalizing symptoms [12] at ages 17, 20, and 24. On a five-point Likert scale, ranging from 1 = *never* to 5 = *very often*, participants were asked to rate items such as crying for no reason or feeling alone. Reliability was high, with values ranging from .85 to .93.

Bullying victimization: For bullying victimization, a validated scale including four items had to be answered on a six-point Likert scale, with 1 = *never* and 6 = *(almost) daily* [13] at all three time points. Cronbach's alpha varied across time points, with values of .69, .63, and .86.

Leisure activities: 12 items were included to measure unstructured leisure activities at age 17, 10 items at age 20, and 7 items at age 24, including the frequency of going out, or meeting friends [8]. Participants answered the items on a six-point Likert scale ranging from 1 = *never* up to 6 = *(almost) daily*. Reliability decreased over time from .79 to .69.

References for Measures

[1] B. B. Quednow, A. Steinhoff, L. Bechtiger, D. Ribeaud, M. Eisner, and L. Shanahan, "High prevalence and early onsets: Legal and illegal substance use in an urban cohort of young adults in Switzerland," *Eur. Addict. Res.*, vol. 28, no. 3, pp. 186–198, 2022, doi: 10.1159/000520178.

[2] F. Vock *et al.*, "Substance use in sexual minority youth: prevalence in an urban cohort," *Child Adolesc. Psychiatry Ment. Health*, vol. 17, no. 1, p. 109, 2023, doi: 10.1186/s13034-023-00657-0.

[3] C. Scholz, J. Cabalzar, T. Kraemer, and M. R. Baumgartner, "A Comprehensive Multi-Analyte Method for Hair Analysis: Substance-Specific Quantification Ranges and Tool for Task-Oriented Data Evaluation," *J. Anal. Toxicol.*, vol. 45, no. 7, pp. 701–712, 2021, doi: 10.1093/jat/bkaa131.

[4] C. Scholz, M. M. Madry, T. Kraemer, and M. R. Baumgartner, "LC–MS-MS Analysis of Δ9-THC, CBN and CBD in Hair: Investigation of Artifacts," *J. Anal. Toxicol.*, vol. 46, no. 5, pp. 504–511, 2022, doi: 10.1093/jat/bkab056.

[5] A. Steinhoff, L. Bechtiger, D. Ribeaud, M. P. Eisner, B. B. Quednow, and L. Shanahan, "Polysubstance Use in Early Adulthood: Patterns and Developmental Precursors in an Urban Cohort," *Front. Behav. Neurosci.*, vol. 15, p. 797473, 2022, doi: 10.3389/fnbeh.2021.797473.

[6] H. B. G. Ganzeboom, P. M. De Graaf, and D. J. Treiman, "A standard international socio-economic index of occupational status," *Soc. Sci. Res.*, vol. 21, no. 1, pp. 1–56, 1992, doi: 10.1016/0049-089X(92)90017-B.

[7] M. P. Marshal *et al.*, "Sexual orientation and adolescent substance use: a meta-analysis and methodological review," *Addiction*, vol. 103, no. 4, pp. 546–556, 2008, doi: 10.1111/j.1360-0443.2008.02149.x.

[8] z-proso Project Team, "z-proso Handbook: Instruments and Procedures in the Adolescent and Young Adult Surveys (Age 11 to 24; Waves K4-K9)," 2024, doi: 10.5167/UZH-253680.

[9] F. D. Alsaker and E. Gutzwiller-Helfenfinger, "Social behavior and peer relationships of victims, bully-victims, and bullies in kindergarten," in *Handbook of School Bullying. An International Perspective*, S. R. Jimerson, S. M. Swearer, and D. L. Espelage, Eds., Mahwah, N.J: Lawrence Erlbaum, 2010, pp. 87–99.

[10] A. L. Murray, M. Eisner, I. Obsuth, and D. Ribeaud, "Identifying Early Markers of 'Late Onset' Attention Deficit and Hyperactivity/Impulsivity Symptoms," *J. Atten. Disord.*, vol. 24, no. 13, pp. 1796–1806, 2020, doi: 10.1177/1087054717705202.

[11] H. G. Grasmick, C. R. Tittle, R. J. Bursik, and B. J. Arneklev, "Testing the Core Empirical Implications of Gottfredson and Hirschi's General Theory of Crime," *J. Res. Crime Delinquency*, vol. 30, no. 1, pp. 5–29, 1993, doi: 10.1177/0022427893030001002.

[12] A. L. Murray, I. Obsuth, M. Eisner, and D. Ribeaud, "Evaluating Longitudinal Invariance in Dimensions of Mental Health Across Adolescence: An Analysis of the Social Behavior Questionnaire," *Assessment*, vol. 26, no. 7, pp. 1234–1245, 2019, doi: 10.1177/1073191117721741.

[13] A. L. Murray, M. Eisner, D. Ribeaud, D. Kaiser, K. McKenzie, and G. Murray, “Validation of a Brief Self-Report Measure of Adolescent Bullying Perpetration and Victimization,” *Assessment*, vol. 28, no. 1, pp. 128–140, 2021, doi: 10.1177/1073191119858406.

Table S1. Descriptives.

Variables	Age	n	Missing n (%)	α	Range	Total n (%) or *M* (*SD*)	Males n (%) or *M* (*SD*)		Female n (%) or *M* (*SD*)	
							HET	SM	HET	SM
Sexual Minority Youth / Young Adults (SM)	17	1,297	–	–	–	147 (11.3%)	605 (92.8%)	47 (7.2%)	545 (84.5%)	100 (15.5%)
	20	1,177	–	–	–	228 (19.4%)	512 (88.1%)	69 (11.9%)	437 (73.3%)	159 (26.7%)
	24	1,158	–	–	–	271 (23.4%)	494 (86.2%)	79 (13.8%)	393 (67.2%)	192 (32.8%)
Socio-economic status (ISEI)*	17	1,232	65 (5.0%)	–	16–99	46.43 (19.43)	46.48 (19.95)	56.09 (16.09)	44.56 (18.57)	51.45 (20.21)
	20	1,118	59 (5.0%)	–	16–99	47.13 (19.69)	46.66 (19.94)	57.18 (18.99)	43.92 (18.97)	52.90 (18.69)
	24	1,106	52 (4.5%)	–	16–99	47.56 (19.47)	47.01 (19.64)	56.94 (18.75)	43.50 (17.99)	53.23 (19.69)
Parental migration background (both parents born abroad)*	17	1,267	30 (2.4%)	–	–	612 (48.3%)	284 (48.2%)	13 (28.3%)	285 (53.4%)	30 (30.6%)
	20	1,177	24 (2.0%)	–	–	550 (47.7%)	246 (49.2%)	23 (34.3%)	238 (55.0%)	43 (28.1%)
	24	1,134	24 (2.1%)	–	–	525 (46.3%)	233 (47.2%)	17 (21.5%)	213 (54.2%)	62 (32.3%)
Education level (higher level)*	17	1,247	50 (3.9%)	–	–	832 (65.6%)	359 (60.3%)	34 (73.9%)	355 (66.9%)	84 (87.5%)
	20	1,133	44 (3.7%)	–	–	781 (68.0%)	313 (62.2%)	53 (77.9%)	283 (66.4%)	132 (86.8%)
	24	1,116	42 (3.6%)	–	–	785 (69.5%)	62.9% (305)	62 (79.5%)	258 (67.2%)	160 (87.4%)
**Covariates**										
Sensation-seeking*	17	1,130	167 (12.9%)	–	0–1	0.57 (0.25)	0.68 (0.22)	0.60 (0.21)	0.47 (0.22)	0.46 (0.27)
	20	1,038	139 (11.8%)	–	0–1	0.57 (0.25)	0.67 (0.22)	0.64 (0.24)	0.46 (0.22)	0.47 (0.25)
	24	1,018	140 (12.1%)	–	0–1	0.57 (0.25)	0.68 (0.22)	0.64 (0.26)	0.47 (0.22)	0.46 (0.25)
Low self-control	17	1,287	10 (0.8%)	.73	1–4	2.22 (0.43)	2.29 (0.42)	2.06 (0.46)	2.15 (0.42)	2.25 (0.38)

	20	1,176	1 (0.1%)	.74	1–4	2.07 (0.42)	2.14 (0.44)	2.03 (0.42)	2.01 (0.40)	2.00 (0.39)
	24	1,156	2 (0.2%)	.74	1–4	1.88 (0.41)	1.94 (0.41)	1.89 (0.43)	1.82 (0.38)	1.85 (0.42)
Internalizing symptoms	17	1,281	16 (1.2%)	.85	1–5	2.28 (0.75)	1.95 (0.58)	2.39 (0.71)	2.51 (0.73)	2.94 (0.81)
	20	1,176	1 (0.1%)	.92	1–5	2.21 (0.76)	1.96 (0.64)	2.31 (0.71)	2.34 (0.78)	2.64 (0.79)
	24	1,156	2 (0.2%)	.93	1–5	2.29 (0.77)	2.07 (0.69)	2.31 (0.67)	2.39 (0.78)	2.64 (0.82)
Bullying victimization	17	1,295	2 (0.2%)	.69	1–6	1.45 (0.57)	1.45 (0.59)	1.75 (0.67)	1.40 (0.52)	1.54 (0.62)
	20	1,176	1 (0.1%)	.63	1–6	1.37 (0.47)	1.36 (0.45)	1.63 (0.61)	1.31 (0.44)	1.46 (0.52)
	24	1,157	1 (0.1%)	.86	1–6	1.46 (0.49)	1.39 (0.44)	1.55 (0.56)	1.45 (0.47)	1.61 (0.57)
Unstructured Leisure activities	17	1,296	1 (0.1%)	.80	1–6	2.63 (0.68)	2.70 (0.70)	2.36 (0.70)	2.57 (0.65)	2.63 (0.65)
	20	1,176	1 (0.1%)	.75	1–6	2.61 (0.61)	2.64 (0.64)	2.58 (0.56)	2.57 (0.59)	2.62 (0.55)
	24	1,155	3 (0.1%)	.82	1–6	2.97 (0.74)	2.97 (0.79)	3.14 (0.59)	2.93 (0.68)	2.96 (0.77)
Exposure to friends' substance use	17	1,206	91 (7.0%)	–	0–1	59.9% (772)	342 (62.9%)	27 (64.3%)	275 (52.5%)	78 (81.2%)
	20	1,123	54 (4.6%)	–	0–1	63.4% (712)	313 (65.5%)	54 (83.1%)	220 (51.6%)	125 (81.2%)
	24	1,089	69 (6.0%)	–	0–1	51.3% (429)	237 (52.0%)	52 (70.3%)	147 (39.0%)	102 (56.0%)

Note. *Only measured at one specific time-point. Please refer to the Methods section.

Table S2a. Baseline models without interaction terms

Outcome	Predictor	Estimate	Standard Error	t-value	p-value	CI LL	CI UL
**Quadratic Regressions**							
	(Intercept)	0.77	0.02	46.64	**<.001*** **	0.73	0.80
Tobacco	Age	-0.01	0.01	-1.89	0.06	-0.02	0.00
	Age^2	-0.03	0.01	-3.51	**<.001*** **	-0.04	-0.01

Outcome	Predictor	Estimate	Standard Error	t-value	p-value	CI LL	CI UL
	Sexuality (ref. HET)	0.00	0.02	0.24	0.81	-0.03	0.04
	Sex (ref. males)	-0.01	0.02	-0.65	0.52	-0.05	0.03
Alcohol	(Intercept)	0.90	0.01	71.54	**<.001*****	0.87	0.92
	Age	0.03	0.00	7.62	**<.001*****	0.03	0.04
	Age^2	-0.03	0.01	-4.16	**<.001*****	-0.04	-0.01
	Sexuality (ref. HET)	0.05	0.02	3.52	**<.001*****	0.02	0.09
	Sex (ref. males)	-0.03	0.02	-2.18	**0.03***	-0.06	0.00
Cannabis	(Intercept)	0.60	0.02	33.27	**<.001*****	0.57	0.64
	Age	-0.02	0.01	-3.55	**<.001*****	-0.04	-0.01
	Age^2	-0.04	0.01	-4.47	**<.001*****	-0.06	-0.02
	Sexuality (ref. HET)	0.12	0.02	5.31	**<.001*****	0.08	0.17
	Sex (ref. males)	-0.14	0.02	-6.49	**<.001*****	-0.18	-0.10
Ecstasy	(Intercept)	0.14	0.01	13.29	**<.001*****	0.12	0.16
	Age	0.03	0.00	6.99	**<.001*****	0.02	0.04
	Age^2	-0.03	0.01	-4.77	**<.001*****	-0.04	-0.02
	Sexuality (ref. HET)	0.09	0.01	6.26	**<.001*****	0.06	0.11
	Sex (ref. males)	-0.06	0.01	-4.68	**<.001*****	-0.08	-0.03
Stimulants	(Intercept)	1.34	0.03	52.83	**<.001*****	1.29	1.39
	Age	0.07	0.01	7.55	**<.001*****	0.05	0.09
	Age^2	-0.06	0.01	-4.25	**<.001*****	-0.08	-0.03
	Sexuality (ref. HET)	0.14	0.03	4.33	**<.001*****	0.08	0.20
	Sex (ref. males)	-0.17	0.03	-5.65	**<.001*****	-0.23	-0.11
Cocaine	(Intercept)	0.13	0.01	13.34	**<.001*****	0.11	0.15
	Age	0.04	0.00	9.15	**<.001*****	0.03	0.04
	Age^2	-0.02	0.01	-4.34	**<.001*****	-0.04	-0.01
	Sexuality (ref. HET)	0.05	0.01	3.78	**<.001*****	0.02	0.07
	Sex (ref. males)	-0.06	0.01	-5.19	**<.001*****	-0.08	-0.04
(Meth-) Amphetamines	(Intercept)	0.08	0.01	9.92	**<.001*****	0.06	0.09
	Age	0.01	0.00	1.69	0.09	0.00	0.01
	Age^2	-0.02	0.00	-4.21	**<.001*****	-0.03	-0.01
	Sexuality (ref. HET)	0.06	0.01	5.75	**<.001*****	0.04	0.08
	Sex (ref. males)	-0.04	0.01	-3.96	**<.001*****	-0.05	-0.02
Hallucinogens	(Intercept)	1.16	0.02	76.77	**<.001*****	1.13	1.19
	Age	0.04	0.01	6.34	**<.001*****	0.03	0.05
	Age^2	-0.02	0.01	-2.45	**0.01***	-0.04	0.00
	Sexuality (ref. HET)	0.13	0.02	6.53	**<.001*****	0.09	0.17
	Sex (ref. males)	-0.10	0.02	-5.81	**<.001*****	-0.13	-0.07
**Linear Regressions**							
Benzodiazepines	(Intercept)	1.07	0.02	56.63	**<.001*****	1.03	1.11

Outcome	Predictor	Estimate	Standard Error	t-value	p-value	CI LL	CI UL
	Age	0.03	0.01	2.02	**.044***	0.00	0.05
	Sexuality (ref. HET)	0.04	0.03	1.57	.116	-0.01	0.10
	Sex (ref. males)	0.04	0.02	1.62	.105	-0.01	0.09
Opioids	(Intercept)	1.24	0.03	48.22	**<.001*****	1.19	1.29
	Age	0.04	0.02	2.09	**.037***	0.00	0.08
	Sexuality (ref. HET)	-0.06	0.04	-1.47	.142	-0.13	0.02
	Sex (ref. males)	0.06	0.03	1.74	.082	-0.01	0.12
**Logistic Regressions (Polysubstance use)**							
**Quadratic Regressions**							
Poly 1	(Intercept)	3.16	0.27	11.68	**<.001*****	2.63	3.69
	Age	0.05	0.06	0.95	0.34	-0.06	0.16
	Age^2	-0.42	0.09	-4.93	**<.001*****	-0.59	-0.25
	Sexuality (ref. HET)	0.76	0.22	3.40	**<.001*****	0.32	1.19
	Sex (ref. males)	-0.84	0.22	-3.78	**<.001*****	-1.28	-0.41
Poly 2	(Intercept)	-5.93	0.42	-14.12	**<.001*****	-6.76	-5.11
	Age	1.20	0.12	10.38	**<.001*****	0.97	1.42
	Age^2	-0.90	0.13	-7.01	**<.001*****	-1.15	-0.65
	Sexuality (ref. HET)	0.93	0.29	3.15	**0.00****	0.35	1.50
	Sex (ref. males)	-1.10	0.33	-3.33	**<.001*****	-1.75	-0.45
**Linear Regression**							
Poly 3	(Intercept)	-0.68	0.12	-5.45	**<.001*****	-0.92	-0.44
	Age	-0.02	0.08	-0.29	0.77	-0.18	0.14
	Sexuality (ref. HET)	1.02	0.18	5.57	**<.001*****	0.66	1.38
	Sex (ref. males)	-1.01	0.17	-6.04	**<.001*****	-1.34	-0.68

Note: For substances available at only two time points (benzodiazepines, opioids, and poly 3), linear regression was used. For the other substances, quadratic regression models were applied. Age2 = quadratic term.

Table S2b. Baseline models with interaction terms

Outcome	Predictor	Estimate	Standard Error	t-value	p-value	CI LL	CI UL
**Quadratic Regression**							

Outcome	Predictor	Estimate	Standard Error	t-value	p-value	CI LL	CI UL
Tobacco	(Intercept)	0.77	0.02	46.53	**<.001*** **	0.74	0.81
	Age	-0.01	0.01	-1.96	**0.05***	-0.02	0.00
	Age^2	-0.03	0.01	-3.51	**<.001*** **	-0.04	-0.01
	Sexuality (ref. HET)	-0.07	0.03	-2.18	**0.03***	-0.14	-0.01
	Sex (ref. males)	-0.03	0.02	-1.49	0.14	-0.07	0.01
	Sexuality*Sex	0.12	0.04	2.89	**0.00****	0.04	0.20
Alcohol	(Intercept)	0.90	0.01	70.90	**<.001*** **	0.88	0.93
	Age	0.03	0.00	7.57	**<.001*** **	0.02	0.04
	Age^2	-0.03	0.01	-4.17	**<.001*** **	-0.04	-0.01
	Sexuality (ref. HET)	0.01	0.03	0.24	0.81	-0.05	0.06
	Sex (ref. males)	-0.04	0.02	-2.80	**0.01****	-0.08	-0.01
	Sexuality*Sex	0.07	0.03	2.31	**0.02***	0.01	0.14
Cannabis	(Intercept)	0.62	0.02	33.57	**<.001*** **	0.58	0.65
	Age	-0.02	0.01	-3.65	**<.001*** **	-0.04	-0.01
	Age^2	-0.04	0.01	-4.48	**<.001*** **	-0.06	-0.02
	Sexuality (ref. HET)	0.00	0.04	-0.13	0.90	-0.08	0.07
	Sex (ref. males)	-0.17	0.02	-7.47	**<.001*** **	-0.22	-0.13
	Sexuality*Sex	0.19	0.05	4.09	**<.001*** **	0.10	0.28
Ecstasy	(Intercept)	0.14	0.01	13.45	**<.001*** **	0.12	0.16
	Age	0.03	0.00	6.94	**<.001*** **	0.02	0.04
	Age^2	-0.03	0.01	-4.77	**<.001*** **	-0.04	-0.02
	Sexuality (ref. HET)	0.05	0.02	2.06	**0.04***	0.00	0.09
	Sex (ref. males)	-0.06	0.01	-5.10	**<.001*** **	-0.09	-0.04
	Sexuality*Sex	0.06	0.03	2.04	**0.04***	0.00	0.11
Stimulants	(Intercept)	1.34	0.03	52.07	**<.001*** **	1.29	1.39
	Age	0.07	0.01	7.53	**<.001*** **	0.05	0.09
	Age^2	-0.06	0.01	-4.25	**<.001*** **	-0.08	-0.03
	Sexuality (ref. HET)	0.12	0.05	2.16	**0.03***	0.01	0.22
	Sex (ref. males)	-0.17	0.03	-5.51	**<.001*** **	-0.24	-0.11
	Sexuality*Sex	0.03	0.07	0.50	0.62	-0.10	0.16
Cocaine	(Intercept)	0.14	0.01	13.29	**<.001*** **	0.12	0.16
	Age	0.04	0.00	9.12	**<.001*** **	0.03	0.04
	Age^2	-0.02	0.01	-4.34	**<.001*** **	-0.04	-0.01
	Sexuality (ref. HET)	0.03	0.02	1.48	0.14	-0.01	0.08
	Sex (ref. males)	-0.06	0.01	-5.21	**<.001*** **	-0.09	-0.04
	Sexuality*Sex	0.03	0.03	0.94	0.35	-0.03	0.08
(Meth-) Amphetamines	(Intercept)	0.08	0.01	9.67	**<.001*** **	0.06	0.09
	Age	0.01	0.00	1.70	0.09	0.00	0.01
	Age^2	-0.02	0.00	-4.21	**<.001*** **	-0.03	-0.01

Outcome	Predictor	Estimate	Standard Error	t-value	p-value	CI LL	CI UL
	Sexuality (ref. HET)	0.06	0.02	3.70	**<.001*** **	0.03	0.10
	Sex (ref. males)	-0.03	0.01	-3.58	**<.001*** **	-0.05	-0.02
	Sexuality*Sex	-0.01	0.02	-0.38	0.71	-0.05	0.03
Hallucinogens	(Intercept)	1.16	0.02	75.34	**<.001*** **	1.13	1.19
	Age	0.04	0.01	6.35	**<.001*** **	0.03	0.05
	Age^2	-0.02	0.01	-2.45	**0.01***	-0.04	0.00
	Sexuality (ref. HET)	0.14	0.03	4.30	**<.001*** **	0.08	0.21
	Sex (ref. males)	-0.10	0.02	-5.26	**<.001*** **	-0.13	-0.06
	Sexuality*Sex	-0.02	0.04	-0.55	0.58	-0.10	0.06
**Linear Regressions**							
Benzodiazepines	(Intercept)	1.07	0.02	54.62	**<.001*** **	1.03	1.11
	Age	0.03	0.01	2.02	**.04***	0.00	0.05
	Sexuality (ref. HET)	0.05	0.05	1.01	.31	-0.05	0.14
	Sex (ref. males)	0.04	0.03	1.52	.13	-0.01	0.09
	Sexuality*Sex	-0.01	0.06	-0.11	.91	-0.12	0.11
Opioids	(Intercept)	1.25	0.03	46.66	**<.001*** **	1.20	1.30
	Age	0.04	0.02	2.08	**.04***	0.00	0.08
	Sexuality (ref. HET)	-0.09	0.07	-1.29	.20	-0.22	0.04
	Sex (ref. males)	0.05	0.04	1.35	.18	-0.02	0.12
	Sexuality*Sex	0.04	0.08	0.53	.60	-0.12	0.20
**Logistic Regressions (Polysubstance use)**							
**Quadratic Regressions**							
Poly 1	(Intercept)	3.27	0.28	11.86	**<.001*** **	2.73	3.81
	Age^2	0.05	0.06	0.83	0.41	-0.06	0.16
	Age	-0.43	0.09	-4.97	**<.001*** **	-0.59	-0.26
	Sexuality (ref. HET)	-0.30	0.37	-0.82	0.41	-1.03	0.42
	Sex (ref. males)	-1.07	0.23	-4.56	**<.001*** **	-1.52	-0.61
	Sexuality*Sex	1.62	0.46	3.51	**<.001*** **	0.72	2.52
Poly 2	(Intercept)	-5.83	0.42	-13.90	**<.001*** **	-6.66	-5.01
	Age^2	1.20	0.12	10.36	**<.001*** **	0.97	1.42
	Age	-0.90	0.13	-7.02	**<.001*** **	-1.15	-0.65
	Sexuality (ref. HET)	0.09	0.45	0.21	0.84	-0.79	0.98
	Sex (ref. males)	-1.42	0.36	-3.90	**<.001*** **	-2.13	-0.71
	Sexuality*Sex	1.42	0.59	2.40	**0.02***	0.26	2.59
**Linear Regression**							
Poly 3	(Intercept)	-0.60	0.13	-4.67	**<.001*** **	-0.85	-0.35
	Age	-0.03	0.08	-0.34	0.73	-0.19	0.13
	Sexuality (ref. HET)	0.38	0.30	1.27	0.20	-0.21	0.97
	Sex (ref. males)	-1.21	0.19	-6.48	**<.001*** **	-1.57	-0.84

Outcome	Predictor	Estimate	Standard Error	t-value	p-value	CI LL	CI UL
	Sexuality*Sex	0.99	0.38	2.63	**0.01****	0.25	1.73

Note: For substances available at only two time points (benzodiazepines, opioids, and poly 3), linear regression was used. For the other substances, quadratic regression models were applied. Age2 = quadratic term.

Table S3a. Sociodemographic models without interaction terms

Outcome	Predictor	Estimate	Standard Error	t-value	p-value	CI LL	CI UL
**Quadratic Regressions**							
Tobacco	(Intercept)	0.74	0.03	25.35	**<.001*****	0.68	0.80
	Age	-0.01	0.01	-2.32	**0.02***	-0.03	0.00
	Age^2	-0.03	0.01	-3.06	**0.00****	-0.04	-0.01
	Sexuality (ref. HET)	0.02	0.02	0.89	0.37	-0.02	0.06
	Sex (ref. males)	0.00	0.02	-0.19	0.85	-0.05	0.04
	Mig. background (ref. natives)	0.02	0.02	1.04	0.30	-0.02	0.07
	ISEI (ref. below median)	-0.02	0.03	-0.76	0.45	-0.07	0.03
	Education (ref. higher education)	0.06	0.02	2.31	**0.02***	0.01	0.11
Alcohol	(Intercept)	0.96	0.02	46.75	**<.001*****	0.92	1.00
	Age	0.03	0.00	7.27	**<.001*****	0.02	0.04
	Age^2	-0.02	0.01	-3.87	**<.001*****	-0.04	-0.01
	Sexuality (ref. HET)	0.03	0.02	1.79	0.07	0.00	0.06
	Sex (ref. males)	-0.04	0.01	-2.84	**0.00****	-0.07	-0.01
	Mig. background (ref. natives)	-0.08	0.02	-4.90	**<.001*****	-0.11	-0.05
	ISEI (ref. below median)	0.05	0.02	2.99	**0.00****	0.02	0.09
	Education (ref. higher education)	-0.11	0.02	-6.16	**<.001*****	-0.14	-0.07
Cannabis	(Intercept)	0.63	0.03	20.27	**<.001*****	0.57	0.69
	Age	-0.03	0.01	-3.91	**<.001*****	-0.04	-0.01
	Age^2	-0.04	0.01	-4.12	**<.001*****	-0.06	-0.02
	Sexuality (ref. HET)	0.09	0.02	3.55	**<.001*****	0.04	0.13
	Sex (ref. males)	-0.14	0.02	-6.15	**<.001*****	-0.18	-0.09
	Mig. background (ref. natives)	-0.09	0.03	-3.70	**<.001*****	-0.14	-0.04
	ISEI (ref. below median)	0.08	0.03	2.92	**0.00****	0.03	0.13

Outcome	Predictor	Estimate	Standard Error	t-value	p-value	CI LL	CI UL
	Education (ref. higher education)	-0.07	0.03	-2.78	**0.01****	-0.12	-0.02
Ecstasy	(Intercept)	0.16	0.02	9.13	**<.001*****	0.13	0.20
	Age	0.03	0.00	6.34	**<.001*****	0.02	0.04
	Age^2	-0.03	0.01	-5.23	**<.001*****	-0.05	-0.02
	Sexuality (ref. HET)	0.09	0.01	5.84	**<.001*****	0.06	0.11
	Sex (ref. males)	-0.05	0.01	-3.91	**<.001*****	-0.07	-0.02
	Mig. background (ref. natives)	-0.06	0.01	-3.99	**<.001*****	-0.08	-0.03
	ISEI (ref. below median)	0.00	0.01	-0.06	0.95	-0.03	0.03
	Education (ref. higher education)	0.02	0.01	1.08	0.28	-0.01	0.04
Stimulants	(Intercept)	1.36	0.04	31.29	**<.001*****	1.28	1.45
	Age	0.07	0.01	7.37	**<.001*****	0.05	0.09
	Age^2	-0.07	0.01	-4.63	**<.001*****	-0.09	-0.04
	Sexuality (ref. HET)	0.15	0.03	4.39	**<.001*****	0.08	0.22
	Sex (ref. males)	-0.16	0.03	-5.10	**<.001*****	-0.22	-0.10
	Mig. background (ref. natives)	-0.11	0.04	-3.07	**0.00****	-0.18	-0.04
	ISEI (ref. below median)	0.00	0.04	-0.07	0.94	-0.08	0.07
	Education (ref. higher education)	0.07	0.04	2.03	**0.04***	0.00	0.15
Cocaine	(Intercept)	0.14	0.02	8.30	**<.001*****	0.11	0.17
	Age	0.04	0.00	8.93	**<.001*****	0.03	0.05
	Age^2	-0.03	0.01	-4.74	**<.001*****	-0.04	-0.02
	Sexuality (ref. HET)	0.05	0.01	3.70	**<.001*****	0.02	0.08
	Sex (ref. males)	-0.05	0.01	-4.58	**<.001*****	-0.08	-0.03
	Mig. background (ref. natives)	-0.04	0.01	-3.06	**0.00****	-0.07	-0.01
	ISEI (ref. below median)	0.00	0.01	0.11	0.91	-0.03	0.03
	Education (ref. higher education)	0.03	0.01	2.42	**0.02***	0.01	0.06
(Meth-) Amphetamines	(Intercept)	0.09	0.01	6.88	**<.001*****	0.07	0.12
	Age	0.01	0.00	1.55	0.12	0.00	0.01
	Age^2	-0.02	0.00	-4.78	**<.001*****	-0.03	-0.01
	Sexuality (ref. HET)	0.06	0.01	5.43	**<.001*****	0.04	0.08
	Sex (ref. males)	-0.03	0.01	-3.68	**<.001*****	-0.05	-0.02
	Mig. background (ref. natives)	-0.02	0.01	-2.28	**0.02***	-0.05	0.00
	ISEI (ref. below median)	0.00	0.01	0.20	0.84	-0.02	0.02
	Education (ref. higher education)	0.00	0.01	-0.11	0.91	-0.02	0.02
Hallucinogens	(Intercept)	1.19	0.03	45.88	**<.001*****	1.14	1.24
	Age	0.04	0.01	5.68	**<.001*****	0.02	0.05
	Age^2	-0.03	0.01	-3.02	**0.00****	-0.05	-0.01
	Sexuality (ref. HET)	0.13	0.02	6.09	**<.001*****	0.09	0.17
	Sex (ref. males)	-0.10	0.02	-5.55	**<.001*****	-0.14	-0.07
	Mig. background (ref. natives)	-0.05	0.02	-2.52	**0.01***	-0.09	-0.01

Outcome	Predictor	Estimate	Standard Error	t-value	p-value	CI LL	CI UL
	ISEI (ref. below median)	0.01	0.02	0.37	0.71	-0.03	0.05
	Education (ref. higher education)	-0.02	0.02	-0.74	0.46	-0.06	0.03
**Linear Regressions**							
	(Intercept)	1.05	0.03	30.54	**<.001*** **	0.98	1.12
	Age	0.02	0.01	1.59	0.11	-0.01	0.05
	Sexuality (ref. HET)	0.05	0.03	1.67	0.10	-0.01	0.11
Benzodiazepines	Sex (ref. males)	0.04	0.03	1.43	0.15	-0.01	0.09
	Mig. background (ref. natives)	0.01	0.03	0.29	0.77	-0.05	0.06
	ISEI (ref. below median)	0.01	0.03	0.17	0.86	-0.05	0.06
	Education (ref. higher education)	0.04	0.03	1.21	0.23	-0.02	0.10
	(Intercept)	1.25	0.05	27.08	**<.001*** **	1.16	1.34
	Age	0.03	0.02	1.57	0.12	-0.01	0.07
	Sexuality (ref. HET)	-0.03	0.04	-0.72	0.47	-0.11	0.05
Opioids	Sex (ref. males)	0.05	0.03	1.54	0.12	-0.01	0.12
	Mig. background (ref. natives)	0.01	0.04	0.18	0.85	-0.07	0.08
	ISEI (ref. below median)	-0.06	0.04	-1.62	0.11	-0.14	0.01
	Education (ref. higher education)	0.06	0.04	1.57	0.12	-0.02	0.14
**Logistic Regressions (Polysubstance use)**							
**Quadratic Regressions**							
	(Intercept)	3.39	0.39	8.67	**<.001*** **	2.62	4.16
	Age	0.03	0.06	0.46	0.65	-0.09	0.14
	Age^2	-0.39	0.09	-4.39	**<.001*** **	-0.57	-0.22
Poly 1	Sexuality (ref. HET)	0.60	0.24	2.54	**0.01***	0.14	1.06
	Sex (ref. males)	-0.86	0.23	-3.70	**<.001*** **	-1.32	-0.41
	Mig. background (ref. natives)	-0.50	0.26	-1.90	0.06	-1.02	0.02
	ISEI (ref. below median)	0.35	0.27	1.27	0.20	-0.19	0.88
	Education (ref. higher education)	-0.34	0.27	-1.28	0.20	-0.87	0.18
	(Intercept)	-5.34	0.64	-8.40	**<.001*** **	-6.58	-4.09
	Age	1.22	0.13	9.61	**<.001*** **	0.97	1.47
	Age^2	-0.99	0.14	-7.15	**<.001*** **	-1.26	-0.72
Poly 2	Sexuality (ref. HET)	0.98	0.32	3.06	**0.00** **	0.35	1.61
	Sex (ref. males)	-1.19	0.36	-3.32	**<.001*** **	-1.90	-0.49
	Mig. background (ref. natives)	-0.99	0.40	-2.45	**0.01***	-1.78	-0.20
	ISEI (ref. below median)	0.12	0.42	0.30	0.77	-0.70	0.95
	Education (ref. higher education)	0.21	0.42	0.49	0.62	-0.61	1.02
**Linear Regression**							
	(Intercept)	-0.51	0.23	-2.27	**0.02***	-0.96	-0.07
Poly3	Age	-0.09	0.09	-1.08	0.28	-0.26	0.08
	Sexuality (ref. HET)	0.96	0.20	4.87	**<.001*** **	0.57	1.34

Outcome	Predictor	Estimate	Standard Error	t-value	p-value	CI LL	CI UL
	Sex (ref. males)	-1.00	0.18	-5.68	**<.001*** **	-1.35	-0.66
	Mig. background (ref. natives)	-0.59	0.19	-3.05	**0.00** **	-0.97	-0.21
	ISEI (ref. below median)	0.25	0.20	1.25	0.21	-0.14	0.64
	Education (ref. higher education)	-0.04	0.20	-0.20	0.84	-0.44	0.35

Note: For substances available at only two time points (benzodiazepines, opioids, and poly 3), linear regression was used. For the other substances, quadratic regression models were applied.

Table S3b. Sociodemographic models with interaction terms

Outcome	Predictor	Estimate	Standard Error	t-value	p-value	CI LL	CI UL
**Quadratic Regressions**							
	(Intercept)	0.75	0.03	25.51	**<.001*** **	0.69	0.80
	Age	-0.01	0.01	-2.38	**0.02***	-0.03	0.00
	Age^2	-0.03	0.01	-3.05	**0.00** **	-0.04	-0.01
	Sexuality (ref. HET)	-0.05	0.04	-1.48	0.14	-0.12	0.02
Tobacco	Sex (ref. males)	-0.02	0.02	-0.95	0.34	-0.06	0.02
	Mig. background (ref. natives)	0.03	0.02	1.09	0.28	-0.02	0.07
	ISEI (ref. below median)	-0.02	0.03	-0.69	0.49	-0.07	0.03
	Education (ref. higher education)	0.06	0.02	2.37	**0.02***	0.01	0.11
	Sexuality*Sex	0.11	0.04	2.52	**0.01***	0.02	0.20
	(Intercept)	0.96	0.02	46.72	**<.001*** **	0.92	1.00
	Age	0.03	0.00	7.24	**<.001*** **	0.02	0.04
	Age^2	-0.02	0.01	-3.87	**<.001*** **	-0.04	-0.01
	Sexuality (ref. HET)	0.00	0.03	-0.02	0.99	-0.05	0.05
Alcohol	Sex (ref. males)	-0.05	0.02	-3.12	**0.00** **	-0.08	-0.02
	Mig. background (ref. natives)	-0.08	0.02	-4.88	**<.001*** **	-0.11	-0.05
	ISEI (ref. below median)	0.05	0.02	3.03	**0.00** **	0.02	0.09
	Education (ref. higher education)	-0.11	0.02	-6.13	**<.001*** **	-0.14	-0.07
	Sexuality*Sex	0.04	0.03	1.37	0.17	-0.02	0.11

Outcome	Predictor	Estimate	Standard Error	t-value	p-value	CI LL	CI UL
Cannabis	(Intercept)	0.64	0.03	20.55	**<.001*** **	0.58	0.70
	Age	-0.03	0.01	-3.99	**<.001*** **	-0.04	-0.01
	Age^2	-0.04	0.01	-4.10	**<.001*** **	-0.06	-0.02
	Sexuality (ref. HET)	-0.04	0.04	-0.87	0.38	-0.11	0.04
	Sex (ref. males)	-0.17	0.02	-7.04	**<.001*** **	-0.21	-0.12
	Mig. background (ref. natives)	-0.09	0.03	-3.64	**<.001*** **	-0.14	-0.04
	ISEI (ref. below median)	0.08	0.03	3.05	**0.00** **	0.03	0.13
	Education (ref. higher education)	-0.07	0.03	-2.70	**0.01** **	-0.12	-0.02
	Sexuality*Sex	0.19	0.05	3.76	**<.001*** **	0.09	0.28
Ecstasy	(Intercept)	0.16	0.02	9.22	**<.001*** **	0.13	0.20
	Age	0.03	0.00	6.31	**<.001*** **	0.02	0.04
	Age^2	-0.03	0.01	-5.22	**<.001*** **	-0.05	-0.02
	Sexuality (ref. HET)	0.06	0.02	2.35	**0.02***	0.01	0.11
	Sex (ref. males)	-0.06	0.01	-4.17	**<.001*** **	-0.08	-0.03
	Mig. background (ref. natives)	-0.06	0.01	-3.96	**<.001*** **	-0.08	-0.03
	ISEI (ref. below median)	0.00	0.01	-0.01	0.99	-0.03	0.03
	Education (ref. higher education)	0.02	0.01	1.11	0.27	-0.01	0.05
	Sexuality*Sex	0.04	0.03	1.44	0.15	-0.02	0.10
Stimulants	(Intercept)	1.36	0.04	31.18	**<.001*** **	1.28	1.45
	Age	0.07	0.01	7.37	**<.001*** **	0.05	0.09
	Age^2	-0.07	0.01	-4.63	**<.001*** **	-0.09	-0.04
	Sexuality (ref. HET)	0.15	0.06	2.68	**0.01** **	0.04	0.27
	Sex (ref. males)	-0.16	0.03	-4.80	**<.001*** **	-0.22	-0.09
	Mig. background (ref. natives)	-0.11	0.04	-3.07	**0.00** **	-0.18	-0.04
	ISEI (ref. below median)	0.00	0.04	-0.08	0.94	-0.08	0.07
	Education (ref. higher education)	0.07	0.04	2.03	**0.04***	0.00	0.15
	Sexuality*Sex	0.00	0.07	-0.06	0.95	-0.14	0.13
Cocaine	(Intercept)	0.14	0.02	8.32	**<.001*** **	0.11	0.17
	Age	0.04	0.00	8.91	**<.001*** **	0.03	0.05
	Age^2	-0.03	0.01	-4.73	**<.001*** **	-0.04	-0.02
	Sexuality (ref. HET)	0.04	0.02	1.83	0.07	0.00	0.09
	Sex (ref. males)	-0.06	0.01	-4.47	**<.001*** **	-0.08	-0.03
	Mig. background (ref. natives)	-0.04	0.01	-3.05	**0.00** **	-0.07	-0.01
	ISEI (ref. below median)	0.00	0.01	0.13	0.90	-0.03	0.03
	Education (ref. higher education)	0.03	0.01	2.43	**0.02***	0.01	0.06
	Sexuality*Sex	0.01	0.03	0.49	0.63	-0.04	0.07
(Meth-) Amphetamines	(Intercept)	0.09	0.01	6.79	**<.001*** **	0.07	0.12
	Age	0.01	0.00	1.56	0.12	0.00	0.01
	Age^2	-0.02	0.00	-4.78	**<.001*** **	-0.03	-0.01

Outcome	Predictor	Estimate	Standard Error	t-value	p-value	CI LL	CI UL
	Sexuality (ref. HET)	0.07	0.02	3.83	**<.001***	0.03	0.11
	Sex (ref. males)	-0.03	0.01	-3.21	**0.00**	-0.05	-0.01
	Mig. background (ref. natives)	-0.02	0.01	-2.29	**0.02***	-0.05	0.00
	ISEI (ref. below median)	0.00	0.01	0.17	0.86	-0.02	0.02
	Education (ref. higher education)	0.00	0.01	-0.13	0.90	-0.02	0.02
	Sexuality*Sex	-0.02	0.02	-0.72	0.47	-0.06	0.03
Hallucinogens	(Intercept)	1.19	0.03	45.63	**<.001***	1.14	1.24
	Age	0.04	0.01	5.70	**<.001***	0.02	0.05
	Age^2	-0.03	0.01	-3.02	**0.00**	-0.05	-0.01
	Sexuality (ref. HET)	0.16	0.04	4.52	**<.001***	0.09	0.23
	Sex (ref. males)	-0.09	0.02	-4.84	**<.001***	-0.13	-0.06
	Mig. background (ref. natives)	-0.05	0.02	-2.54	**0.01***	-0.09	-0.01
	ISEI (ref. below median)	0.01	0.02	0.33	0.74	-0.04	0.05
	Education (ref. higher education)	-0.02	0.02	-0.77	0.44	-0.06	0.03
	Sexuality*Sex	-0.05	0.04	-1.09	0.27	-0.13	0.04
Linear Regressions							
Benzodiazepines	(Intercept)	1.05	0.03	30.29	**<.001***	0.98	1.12
	Age	0.02	0.01	1.59	0.11	-0.01	0.05
	Sexuality (ref. HET)	0.06	0.05	1.25	0.21	-0.04	0.16
	Sex (ref. males)	0.04	0.03	1.43	0.15	-0.01	0.09
	Mig. background (ref. natives)	0.01	0.03	0.29	0.78	-0.05	0.06
	ISEI (ref. below median)	0.00	0.03	0.16	0.87	-0.05	0.06
	Education (ref. higher education)	0.04	0.03	1.20	0.23	-0.02	0.10
	Sexuality*Sex	-0.02	0.06	-0.32	0.75	-0.14	0.10
Opioids	(Intercept)	0.03	0.02	1.57	0.12	-0.01	0.07
	Age	-0.04	0.07	-0.55	0.58	-0.17	0.10
	Sexuality (ref. HET)	0.05	0.04	1.33	0.18	-0.02	0.12
	Sex (ref. males)	0.01	0.04	0.19	0.85	-0.07	0.08
	Mig. background (ref. natives)	-0.06	0.04	-1.61	0.11	-0.14	0.01
	ISEI (ref. below median)	0.06	0.04	1.57	0.12	-0.02	0.14
	Education (ref. higher education)	0.01	0.08	0.15	0.88	-0.15	0.18
	Sexuality*Sex	0.03	0.02	1.57	0.12	-0.01	0.07
**Logistic Regressions (Polysubstance use)**							
**Quadratic Regressions**							
Poly 1	(Intercept)	3.47	0.40	8.78	**<.001***	2.70	4.25
	Age	0.02	0.06	0.36	0.72	-0.10	0.14
	Age^2	-0.40	0.09	-4.42	**<.001***	-0.57	-0.22
	Sexuality (ref. HET)	-0.35	0.39	-0.90	0.37	-1.13	0.42
	Sex (ref. males)	-1.06	0.25	-4.35	**<.001***	-1.55	-0.58

Outcome	Predictor	Estimate	Standard Error	t-value	p-value	CI LL	CI UL
	Mig. background (ref. natives)	-0.50	0.26	-1.87	0.06	-1.01	0.02
	ISEI (ref. below median)	0.37	0.27	1.36	0.17	-0.16	0.91
	Education (ref. higher education)	-0.33	0.27	-1.22	0.22	-0.85	0.20
	Sexuality*Sex	1.44	0.49	2.96	**0.00****	0.49	2.39
Poly 2	(Intercept)	-5.30	0.64	-8.32	**<.001*****	-6.54	-4.05
	Age	1.22	0.13	9.60	**<.001*****	0.97	1.47
	Age^2	-0.99	0.14	-7.15	**<.001*****	-1.26	-0.72
	Sexuality (ref. HET)	0.35	0.48	0.73	0.46	-0.59	1.30
	Sex (ref. males)	-1.43	0.39	-3.67	**<.001*****	-2.19	-0.67
	Mig. background (ref. natives)	-0.99	0.41	-2.43	**0.02***	-1.78	-0.19
	ISEI (ref. below median)	0.14	0.42	0.33	0.74	-0.69	0.96
	Education (ref. higher education)	0.23	0.42	0.55	0.58	-0.59	1.05
	Sexuality*Sex	1.09	0.64	1.72	0.09	-0.15	2.34
**Linear Regression**							
Poly3	(Intercept)	-0.46	0.23	-2.02	**0.04***	-0.91	-0.01
	Age	-0.09	0.09	-1.10	0.27	-0.26	0.07
	Sexuality (ref. HET)	0.42	0.32	1.32	0.19	-0.20	1.04
	Sex (ref. males)	-1.17	0.20	-5.97	**<.001*****	-1.55	-0.79
	Mig. background (ref. natives)	-0.58	0.19	-2.99	**0.00****	-0.96	-0.20
	ISEI (ref. below median)	0.26	0.20	1.33	0.18	-0.13	0.65
	Education (ref. higher education)	-0.03	0.20	-0.15	0.88	-0.42	0.37
	Sexuality*Sex	0.84	0.40	2.13	**0.03***	0.07	1.62

Note: For substances available at only two time points (benzodiazepines, opioids, and poly 3), linear regression was used. For the other substances, quadratic regression models were applied.

Table S4a. Full models without interaction terms

Outcome	Predictor	Estimate	Standard Error	t-value	p-value	CI LL	CI UL
**Quadratic Regressions**							
Tobacco	(Intercept)	0.60	0.03	18.48	**<.001*****	0.53	0.66
	Age	-0.01	0.01	-1.39	0.17	-0.02	0.00

Outcome	Predictor	Estimate	Standard Error	t-value	p-value	CI LL	CI UL
	Age^2	-0.01	0.01	-1.61	0.11	-0.03	0.00
	Sexuality (ref. HET)	0.00	0.02	-0.08	0.93	-0.05	0.04
	Sex (ref. males)	0.06	0.02	2.47	**0.01***	0.01	0.10
	Mig. background (ref. natives)	0.05	0.02	2.12	**0.03***	0.00	0.10
	ISEI (ref. below median)	-0.03	0.03	-1.19	0.23	-0.08	0.02
	Education (ref. higher education)	0.07	0.02	2.77	**0.01****	0.02	0.12
	Exposure	0.16	0.02	9.58	**<.001*****	0.13	0.19
	Sensation seeking	0.02	0.01	1.60	0.11	0.00	0.04
	Low self-control	0.05	0.01	4.95	**<.001*****	0.03	0.06
	Inter. Symptoms	0.00	0.01	-0.39	0.69	-0.02	0.01
	Bullying	0.02	0.01	2.12	**0.03***	0.00	0.03
	Leisure	0.06	0.01	6.96	**<.001*****	0.04	0.08
Alcohol	(Intercept)	0.89	0.02	38.83	**<.001*****	0.84	0.93
	Age	0.03	0.00	6.53	**<.001*****	0.02	0.04
	Age^2	-0.02	0.01	-2.25	**0.02***	-0.03	0.00
	Sexuality (ref. HET)	0.03	0.02	1.66	0.10	0.00	0.06
	Sex (ref. males)	-0.03	0.02	-2.08	**0.04***	-0.07	0.00
	Mig. background (ref. natives)	-0.07	0.02	-4.39	**<.001*****	-0.11	-0.04
	ISEI (ref. below median)	0.04	0.02	2.17	**0.03***	0.00	0.07
	Education (ref. higher education)	-0.10	0.02	-5.60	**<.001*****	-0.13	-0.06
	Exposure	0.10	0.01	8.45	**<.001*****	0.08	0.13
	Sensation seeking	-0.01	0.01	-0.92	0.36	-0.02	0.01
	Low self-control	0.02	0.01	2.78	**0.01****	0.01	0.03
	Inter. Symptoms	0.00	0.01	0.64	0.52	-0.01	0.02
	Bullying	0.00	0.01	-0.31	0.76	-0.01	0.01
	Leisure	0.03	0.01	4.11	**<.001*****	0.01	0.04
Cannabis	(Intercept)	0.33	0.03	10.59	**<.001*****	0.27	0.39
	Age	-0.01	0.01	-0.96	0.34	-0.02	0.01
	Age^2	-0.02	0.01	-1.84	0.07	-0.04	0.00
	Sexuality (ref. HET)	0.05	0.02	2.20	**0.03***	0.01	0.09
	Sex (ref. males)	-0.06	0.02	-3.00	**0.00****	-0.11	-0.02
	Mig. background (ref. natives)	-0.04	0.02	-1.63	0.10	-0.08	0.01
	ISEI (ref. below median)	0.06	0.02	2.47	**0.01***	0.01	0.10
	Education (ref. higher education)	-0.03	0.02	-1.10	0.27	-0.07	0.02
	Exposure	0.41	0.02	23.19	**<.001*****	0.38	0.44
	Sensation seeking	0.04	0.01	3.79	**<.001*****	0.02	0.06
	Low self-control	0.05	0.01	5.13	**<.001*****	0.03	0.07
	Inter. Symptoms	0.03	0.01	3.09	**0.00****	0.01	0.05
	Bullying	0.00	0.01	-0.27	0.78	-0.02	0.01

Outcome	Predictor	Estimate	Standard Error	t-value	p-value	CI LL	CI UL
	Leisure	0.05	0.01	5.97	**<.001*** **	0.04	0.07
Ecstasy	(Intercept)	0.10	0.02	4.66	**<.001*** **	0.06	0.14
	Age	0.04	0.01	7.02	**<.001*** **	0.03	0.05
	Age^2	-0.03	0.01	-4.25	**<.001*** **	-0.04	-0.02
	Sexuality (ref. HET)	0.07	0.02	4.65	**<.001*** **	0.04	0.10
	Sex (ref. males)	-0.02	0.01	-1.07	0.28	-0.04	0.01
	Mig. background (ref. natives)	-0.05	0.01	-3.56	**<.001*** **	-0.08	-0.02
	ISEI (ref. below median)	-0.01	0.02	-0.79	0.43	-0.04	0.02
	Education (ref. higher education)	0.03	0.02	1.80	0.07	0.00	0.06
	Exposure	0.09	0.01	7.20	**<.001*** **	0.06	0.11
	Sensation seeking	0.02	0.01	3.30	**<.001*** **	0.01	0.04
	Low self-control	0.03	0.01	5.28	**<.001*** **	0.02	0.05
	Inter. Symptoms	0.01	0.01	1.22	0.22	0.00	0.02
	Bullying	0.01	0.01	1.62	0.11	0.00	0.02
	Leisure	0.03	0.01	4.79	**<.001*** **	0.02	0.04
Stimulants	(Intercept)	1.23	0.05	24.96	**<.001*** **	1.14	1.33
	Age	0.09	0.01	7.67	**<.001*** **	0.06	0.11
	Age^2	-0.06	0.02	-3.88	**<.001*** **	-0.09	-0.03
	Sexuality (ref. HET)	0.10	0.04	2.89	**0.00** **	0.03	0.17
	Sex (ref. males)	-0.10	0.03	-2.82	**0.00** **	-0.17	-0.03
	Mig. background (ref. natives)	-0.10	0.04	-2.75	**0.01** **	-0.17	-0.03
	ISEI (ref. below median)	-0.03	0.04	-0.87	0.38	-0.11	0.04
	Education (ref. higher education)	0.11	0.04	2.96	**0.00** **	0.04	0.18
	Exposure	0.17	0.03	6.30	**<.001*** **	0.12	0.23
	Sensation seeking	0.05	0.02	2.85	**0.00** **	0.02	0.08
	Low self-control	0.07	0.01	4.97	**<.001*** **	0.04	0.10
	Inter. Symptoms	0.04	0.01	2.46	**0.01***	0.01	0.07
	Bullying	0.04	0.01	2.68	**0.01** **	0.01	0.06
	Leisure	0.06	0.01	4.03	**<.001*** **	0.03	0.08
Cocaine	(Intercept)	0.08	0.02	4.32	**<.001*** **	0.05	0.12
	Age	0.04	0.00	9.05	**<.001*** **	0.03	0.05
	Age^2	-0.02	0.01	-3.46	**<.001*** **	-0.04	-0.01
	Sexuality (ref. HET)	0.03	0.01	2.30	**0.02***	0.00	0.06
	Sex (ref. males)	-0.04	0.01	-2.69	**0.01** **	-0.06	-0.01
	Mig. background (ref. natives)	-0.04	0.01	-2.61	**0.01** **	-0.06	-0.01
	ISEI (ref. below median)	-0.01	0.01	-0.80	0.42	-0.04	0.02
	Education (ref. higher education)	0.05	0.01	3.32	<.001***	0.02	0.08
	Exposure	0.08	0.01	6.88	**<.001*** **	0.06	0.10
	Sensation seeking	0.02	0.01	2.33	**0.02***	0.00	0.03

Outcome	Predictor	Estimate	Standard Error	t-value	p-value	CI LL	CI UL
	Low self-control	0.02	0.01	3.89	**<.001*** **	0.01	0.03
	Inter. Symptoms	0.01	0.01	2.10	**0.04***	0.00	0.02
	Bullying	0.01	0.01	2.48	**0.01***	0.00	0.02
	Leisure	0.02	0.01	3.71	**<.001*** **	0.01	0.03
(Meth-) Amphetamines	(Intercept)	0.07	0.02	4.20	**<.001*** **	0.04	0.10
	Age	0.01	0.00	2.07	**0.04***	0.00	0.02
	Age^2	-0.02	0.01	-4.43	**<.001*** **	-0.03	-0.01
	Sexuality (ref. HET)	0.05	0.01	4.01	**<.001*** **	0.02	0.07
	Sex (ref. males)	-0.02	0.01	-1.56	0.12	-0.04	0.00
	Mig. background (ref. natives)	-0.03	0.01	-2.32	**0.02***	-0.05	0.00
	ISEI (ref. below median)	0.00	0.01	-0.23	0.82	-0.03	0.02
	Education (ref. higher education)	0.00	0.01	0.16	0.87	-0.02	0.02
	Exposure	0.04	0.01	4.01	**<.001*** **	0.02	0.05
	Sensation seeking	0.01	0.01	2.55	**0.01***	0.00	0.02
	Low self-control	0.02	0.00	4.82	**<.001*** **	0.01	0.03
	Inter. Symptoms	0.01	0.00	2.08	**0.04***	0.00	0.02
	Bullying	0.01	0.00	2.13	**0.03***	0.00	0.02
	Leisure	0.02	0.00	3.66	**<.001*** **	0.01	0.03
Hallucinogens	(Intercept)	1.11	0.03	36.75	**<.001*** **	1.05	1.17
	Age	0.04	0.01	6.15	**<.001*** **	0.03	0.06
	Age^2	-0.02	0.01	-2.42	**0.02***	-0.04	0.00
	Sexuality (ref. HET)	0.14	0.02	6.12	**<.001*** **	0.09	0.18
	Sex (ref. males)	-0.05	0.02	-2.56	**0.01***	-0.09	-0.01
	Mig. background (ref. natives)	-0.04	0.02	-1.95	0.05	-0.08	0.00
	ISEI (ref. below median)	0.00	0.02	0.10	0.92	-0.04	0.05
	Education (ref. higher education)	-0.01	0.02	-0.24	0.81	-0.05	0.04
	Exposure	0.08	0.02	4.66	**<.001*** **	0.05	0.11
	Sensation seeking	0.04	0.01	3.72	**<.001*** **	0.02	0.06
	Low self-control	0.04	0.01	4.32	**<.001*** **	0.02	0.06
	Inter. Symptoms	0.01	0.01	0.83	0.41	-0.01	0.03
	Bullying	0.01	0.01	0.82	0.41	-0.01	0.02
	Leisure	0.03	0.01	3.53	**<.001*** **	0.01	0.05
**Linear Regressions**							
Benzodiazepines	(Intercept)	1.06	0.04	24.83	**<.001*** **	0.97	1.14
	Age	0.04	0.02	2.20	**0.03***	0.00	0.07
	Sexuality (ref. HET)	0.00	0.03	-0.05	0.96	-0.07	0.06
	Sex (ref. males)	0.01	0.03	0.34	0.74	-0.05	0.07
	Mig. background (ref. natives)	-0.02	0.03	-0.52	0.60	-0.08	0.04
	ISEI (ref. below median)	-0.01	0.03	-0.24	0.81	-0.07	0.06

Outcome	Predictor	Estimate	Standard Error	t-value	p-value	CI LL	CI UL
	Education (ref. higher education)	0.02	0.03	0.68	0.50	-0.04	0.09
	Exposure	0.07	0.03	2.65	**0.01****	0.02	0.12
	Sensation seeking	0.02	0.01	1.22	0.22	-0.01	0.05
	Low self-control	0.02	0.01	1.34	0.18	-0.01	0.05
	Inter. Symptoms	0.10	0.01	7.14	**<.001*****	0.08	0.13
	Bullying	0.01	0.01	0.73	0.46	-0.02	0.04
	Leisure	0.00	0.01	-0.05	0.96	-0.03	0.03
Opioids	(Intercept)	1.27	0.06	22.85	**<.001*****	1.16	1.38
	Age	0.05	0.02	2.13	**0.03***	0.00	0.09
	Sexuality (ref. HET)	-0.10	0.04	-2.24	**0.03***	-0.18	-0.01
	Sex (ref. males)	0.03	0.04	0.72	0.47	-0.05	0.10
	Mig. background (ref. natives)	-0.02	0.04	-0.43	0.67	-0.10	0.06
	ISEI (ref. below median)	-0.05	0.04	-1.29	0.20	-0.14	0.03
	Education (ref. higher education)	0.06	0.04	1.41	0.16	-0.02	0.14
	Exposure	0.01	0.04	0.37	0.71	-0.06	0.08
	Sensation seeking	0.00	0.02	0.26	0.79	-0.03	0.04
	Low self-control	0.07	0.02	3.93	**<.001*****	0.04	0.11
	Inter. Symptoms	0.11	0.02	5.52	**<.001*****	0.07	0.14
	Bullying	0.03	0.02	1.75	0.08	0.00	0.07
	Leisure	0.01	0.02	0.42	0.67	-0.03	0.04
**Logistic Regressions (Polysubstance use)**							
**Quadratic Regressions**							
Poly 1	(Intercept)	0.95	0.33	2.85	**0.00****	0.30	1.60
	Age	0.09	0.07	1.32	0.19	-0.05	0.23
	Age^2	-0.24	0.10	-2.35	**0.02***	-0.44	-0.04
	Sexuality (ref. HET)	0.42	0.25	1.68	0.09	-0.07	0.90
	Sex (ref. males)	-0.06	0.23	-0.25	0.81	-0.51	0.40
	Mig. background (ref. natives)	-0.10	0.24	-0.42	0.68	-0.58	0.38
	ISEI (ref. below median)	0.23	0.25	0.89	0.37	-0.27	0.72
	Education (ref. higher education)	0.00	0.24	0.00	1.00	-0.48	0.48
	Exposure	2.43	0.19	12.85	**<.001*****	2.06	2.80
	Sensation seeking	0.24	0.11	2.14	**0.03***	0.02	0.46
	Low self-control	0.53	0.10	5.29	**<.001*****	0.33	0.73
	Inter. Symptoms	0.07	0.10	0.72	0.47	-0.12	0.27
	Bullying	0.09	0.09	0.97	0.33	-0.09	0.27
	Leisure	0.53	0.09	5.82	**<.001*****	0.35	0.71
Poly 2	(Intercept)	-3.98	0.39	-10.12	**<.001*****	-4.75	-3.21
	Age	0.97	0.11	9.22	**<.001*****	0.76	1.18
	Age^2	-0.57	0.12	-4.94	**<.001*****	-0.80	-0.35

Outcome	Predictor	Estimate	Standard Error	t-value	p-value	CI LL	CI UL
	Sexuality (ref. HET)	0.81	0.23	3.56	**<.001*** **	0.37	1.26
	Sex (ref. males)	-0.87	0.24	-3.62	**<.001*** **	-1.34	-0.40
	Mig. background (ref. natives)	-0.81	0.24	-3.30	**<.001*** **	-1.29	-0.33
	ISEI (ref. below median)	0.21	0.25	0.83	0.41	-0.28	0.70
	Education (ref. higher education)	0.32	0.26	1.25	0.21	-0.18	0.82
	Exposure	2.41	0.26	9.20	**<.001*** **	1.90	2.92
	Sensation seeking	0.32	0.11	2.80	**0.01** **	0.10	0.54
	Low self-control	0.62	0.10	6.00	**<.001*** **	0.42	0.82
	Inter. Symptoms	0.27	0.10	2.59	**0.01** **	0.06	0.47
	Bullying	0.00	0.09	0.05	0.96	-0.18	0.19
	Leisure	0.57	0.10	5.50	**<.001*** **	0.36	0.77
**Linear Regression**							
	(Intercept)	-1.81	0.28	-6.44	**<.001*** **	-2.37	-1.26
	Age	0.14	0.10	1.37	0.17	-0.06	0.33
	Sexuality (ref. HET)	0.55	0.20	2.75	**0.01** **	0.16	0.95
	Sex (ref. males)	-0.75	0.20	-3.79	**<.001*** **	-1.13	-0.36
	Mig. background (ref. natives)	-0.44	0.20	-2.18	**0.03***	-0.83	-0.04
	ISEI (ref. below median)	0.22	0.21	1.06	0.29	-0.19	0.63
Poly3	Education (ref. higher education)	0.09	0.21	0.43	0.67	-0.33	0.51
	Exposure	1.86	0.18	10.22	**<.001*** **	1.50	2.22
	Sensation seeking	0.23	0.09	2.44	**0.01***	0.05	0.42
	Low self-control	0.46	0.09	5.10	**<.001*** **	0.28	0.64
	Inter. Symptoms	0.43	0.09	4.65	**<.001*** **	0.25	0.62
	Bullying	0.06	0.08	0.66	0.51	-0.11	0.22
	Leisure	0.33	0.09	3.71	**<.001*** **	0.16	0.51

Note: For substances available at only two time points (benzodiazepines, opioids, and poly 3), linear regression was used. For the other substances, quadratic regression models were applied.

Table S4b. Full models with interaction terms

Outcome	Predictor	Estimate	Standard Error	t-value	p-value	CI LL	CI UL
**Quadratic Regressions**							
Tobacco	(Intercept)	0.60	0.03	18.60	**<.001*** **	0.54	0.67
	Age	-0.01	0.01	-1.44	0.15	-0.02	0.00

Outcome	Predictor	Estimate	Standard Error	t-value	p-value	CI LL	CI UL
	Age^2	-0.01	0.01	-1.61	0.11	-0.03	0.00
	Sexuality (ref. HET)	-0.06	0.04	-1.61	0.11	-0.13	0.01
	Sex (ref. males)	0.04	0.02	1.75	0.08	-0.01	0.09
	Mig. background (ref. natives)	0.05	0.02	2.16	**0.03***	0.00	0.10
	ISEI (ref. below median)	-0.03	0.03	-1.13	0.26	-0.08	0.02
	Education (ref. higher education)	0.07	0.02	2.82	**0.00****	0.02	0.12
	Exposure	0.16	0.02	9.53	**<.001*****	0.13	0.19
	Sensation seeking	0.02	0.01	1.57	0.12	0.00	0.04
	Low self-control	0.04	0.01	4.87	**<.001*****	0.03	0.06
	Inter. Symptoms	0.00	0.01	-0.33	0.74	-0.02	0.02
	Bullying	0.02	0.01	2.16	**0.03***	0.00	0.03
	Leisure	0.06	0.01	7.00	**<.001*****	0.04	0.08
	Sexuality*Sex	0.09	0.05	1.95	0.05	0.00	0.18
Alcohol	(Intercept)	0.89	0.02	38.64	**<.001*****	0.84	0.93
	Age	0.03	0.00	6.53	**<.001*****	0.02	0.04
	Age^2	-0.02	0.01	-2.25	**0.02***	-0.03	0.00
	Sexuality (ref. HET)	0.03	0.03	0.97	0.33	-0.03	0.08
	Sex (ref. males)	-0.03	0.02	-1.99	**0.05***	-0.07	0.00
	Mig. background (ref. natives)	-0.07	0.02	-4.38	**<.001*****	-0.11	-0.04
	ISEI (ref. below median)	0.04	0.02	2.17	**0.03***	0.00	0.07
	Education (ref. higher education)	-0.10	0.02	-5.59	**<.001*****	-0.13	-0.06
	Exposure	0.10	0.01	8.44	**<.001*****	0.08	0.13
	Sensation seeking	-0.01	0.01	-0.92	0.36	-0.02	0.01
	Low self-control	0.02	0.01	2.78	**0.01****	0.01	0.03
	Inter. Symptoms	0.00	0.01	0.64	0.52	-0.01	0.02
	Bullying	0.00	0.01	-0.31	0.76	-0.01	0.01
	Leisure	0.03	0.01	4.11	**<.001*****	0.01	0.04
	Sexuality*Sex	0.00	0.03	0.04	0.97	-0.06	0.07
Cannabis	(Intercept)	0.34	0.03	10.88	**<.001*****	0.28	0.40
	Age	-0.01	0.01	-1.03	0.30	-0.02	0.01
	Age^2	-0.02	0.01	-1.85	0.06	-0.04	0.00
	Sexuality (ref. HET)	-0.04	0.04	-1.14	0.25	-0.12	0.03
	Sex (ref. males)	-0.09	0.02	-3.89	**<.001*****	-0.13	-0.04
	Mig. background (ref. natives)	-0.03	0.02	-1.57	0.12	-0.08	0.01
	ISEI (ref. below median)	0.06	0.02	2.57	**0.01***	0.01	0.10
	Education (ref. higher education)	-0.02	0.02	-1.02	0.31	-0.07	0.02
	Exposure	0.41	0.02	23.15	**<.001*****	0.37	0.44
	Sensation seeking	0.04	0.01	3.75	**<.001*****	0.02	0.06
	Low self-control	0.05	0.01	4.99	**<.001*****	0.03	0.06

Outcome	Predictor	Estimate	Standard Error	t-value	p-value	CI LL	CI UL
	Inter. Symptoms	0.03	0.01	3.18	**0.00****	0.01	0.05
	Bullying	0.00	0.01	-0.18	0.85	-0.02	0.02
	Leisure	0.05	0.01	6.03	**<.001*****	0.04	0.07
	Sexuality*Sex	0.14	0.05	3.10	**0.00****	0.05	0.23
	(Intercept)	0.10	0.02	4.71	**<.001*****	0.06	0.14
	Age	0.04	0.01	7.00	**<.001*****	0.03	0.05
	Age^2	-0.03	0.01	-4.25	**<.001*****	-0.04	-0.02
	Sexuality (ref. HET)	0.06	0.03	2.22	**0.03***	0.01	0.11
	Sex (ref. males)	-0.02	0.02	-1.27	0.21	-0.05	0.01
	Mig. background (ref. natives)	-0.05	0.01	-3.55	**<.001*****	-0.08	-0.02
	ISEI (ref. below median)	-0.01	0.02	-0.76	0.45	-0.04	0.02
Ecstasy	Education (ref. higher education)	0.03	0.02	1.82	**0.07**	0.00	0.06
	Exposure	0.09	0.01	7.18	**<.001*****	0.06	0.11
	Sensation seeking	0.02	0.01	3.29	**0.00****	0.01	0.04
	Low self-control	0.03	0.01	5.24	**<.001*****	0.02	0.05
	Inter. Symptoms	0.01	0.01	1.24	0.22	0.00	0.02
	Bullying	0.01	0.01	1.64	0.10	0.00	0.02
	Leisure	0.03	0.01	4.80	**<.001*****	0.02	0.04
	Sexuality*Sex	0.02	0.03	0.75	0.45	-0.04	0.08
	(Intercept)	1.23	0.05	24.75	**<.001*****	1.13	1.33
	Age	0.09	0.01	7.69	**<.001*****	0.06	0.11
	Age^2	-0.06	0.02	-3.88	**<.001*****	-0.09	-0.03
	Sexuality (ref. HET)	0.14	0.06	2.28	**0.02***	0.02	0.25
	Sex (ref. males)	-0.09	0.04	-2.44	**0.01***	-0.16	-0.02
	Mig. background (ref. natives)	-0.10	0.04	-2.76	**0.01****	-0.17	-0.03
	ISEI (ref. below median)	-0.03	0.04	-0.89	0.37	-0.11	0.04
Stimulants	Education (ref. higher education)	0.11	0.04	2.94	**0.00****	0.04	0.18
	Exposure	0.17	0.03	6.31	**<.001*****	0.12	0.23
	Sensation seeking	0.05	0.02	2.86	**0.00****	0.02	0.08
	Low self-control	0.07	0.01	5.00	**<.001*****	0.04	0.10
	Inter. Symptoms	0.04	0.02	2.44	**0.01***	0.01	0.07
	Bullying	0.04	0.01	2.66	**0.01****	0.01	0.06
	Leisure	0.06	0.01	4.02	**<.001*****	0.03	0.08
	Sexuality*Sex	-0.05	0.07	-0.68	0.50	-0.19	0.09
	(Intercept)	0.08	0.02	4.28	**<.001*****	0.05	0.12
	Age	0.04	0.00	9.05	**<.001*****	0.03	0.05
Cocaine	Age^2	-0.02	0.01	-3.46	**<.001*****	-0.04	-0.01
	Sexuality (ref. HET)	0.04	0.02	1.53	0.13	-0.01	0.08
	Sex (ref. males)	-0.04	0.01	-2.47	**0.01***	-0.06	-0.01

Outcome	Predictor	Estimate	Standard Error	t-value	p-value	CI LL	CI UL
	Mig. background (ref. natives)	-0.04	0.01	-2.61	**0.01****	-0.06	-0.01
	ISEI (ref. below median)	-0.01	0.01	-0.80	0.42	-0.04	0.02
	Education (ref. higher education)	0.05	0.01	3.31	**<.001*****	0.02	0.08
	Exposure	0.08	0.01	6.87	**<.001*****	0.06	0.10
	Sensation seeking	0.02	0.01	2.33	**0.02***	0.00	0.03
	Low self-control	0.02	0.01	3.89	**<.001*****	0.01	0.03
	Inter. Symptoms	0.01	0.01	2.10	**0.04***	0.00	0.02
	Bullying	0.01	0.01	2.48	**0.01***	0.00	0.02
	Leisure	0.02	0.01	3.70	**<.001*****	0.01	0.03
	Sexuality*Sex	-0.01	0.03	-0.17	0.86	-0.06	0.05
(Meth-) Amphetamines	(Intercept)	0.07	0.02	4.10	**<.001*****	0.03	0.10
	Age	0.01	0.00	2.09	**0.04***	0.00	0.02
	Age^2	-0.02	0.01	-4.43	**<.001*****	-0.03	-0.01
	Sexuality (ref. HET)	0.06	0.02	2.98	**0.00****	0.02	0.10
	Sex (ref. males)	-0.01	0.01	-1.22	0.22	-0.04	0.01
	Mig. background (ref. natives)	-0.03	0.01	-2.33	**0.02***	-0.05	0.00
	ISEI (ref. below median)	0.00	0.01	-0.25	0.80	-0.03	0.02
	Education (ref. higher education)	0.00	0.01	0.14	0.89	-0.02	0.02
	Exposure	0.04	0.01	4.03	**<.001*****	0.02	0.05
	Sensation seeking	0.01	0.01	2.56	**0.01***	0.00	0.02
	Low self-control	0.02	0.00	4.85	**<.001*****	0.01	0.03
	Inter. Symptoms	0.01	0.00	2.06	**0.04***	0.00	0.02
	Bullying	0.01	0.00	2.11	**0.03***	0.00	0.02
	Leisure	0.02	0.00	3.65	**<.001*****	0.01	0.03
	Sexuality*Sex	-0.02	0.02	-0.71	0.48	-0.06	0.03
Hallucinogens	(Intercept)	1.10	0.03	36.36	**<.001*****	1.04	1.16
	Age	0.04	0.01	6.20	**<.001*****	0.03	0.06
	Age^2	-0.02	0.01	-2.42	**0.02***	-0.04	0.00
	Sexuality (ref. HET)	0.20	0.04	5.42	**<.001*****	0.13	0.27
	Sex (ref. males)	-0.04	0.02	-1.67	0.10	-0.08	0.01
	Mig. background (ref. natives)	-0.04	0.02	-1.99	**0.05***	-0.09	0.00
	ISEI (ref. below median)	0.00	0.02	0.04	0.97	-0.04	0.04
	Education (ref. higher education)	-0.01	0.02	-0.30	0.76	-0.05	0.04
	Exposure	0.08	0.02	4.72	**<.001*****	0.05	0.12
	Sensation seeking	0.04	0.01	3.75	**<.001*****	0.02	0.06
	Low self-control	0.04	0.01	4.42	**<.001*****	0.02	0.06
	Inter. Symptoms	0.01	0.01	0.77	0.44	-0.01	0.03
	Bullying	0.01	0.01	0.76	0.45	-0.01	0.02
	Leisure	0.03	0.01	3.51	**<.001*****	0.01	0.05

Outcome	Predictor	Estimate	Standard Error	t-value	p-value	CI LL	CI UL
	Sexuality*Sex	-0.10	0.04	-2.16	**0.03***	-0.19	-0.01
**Linear Regressions**							
Benzodiazepines	(Intercept)	1.06	0.04	24.66	**<.001*****	0.98	1.14
	Age	0.04	0.02	2.20	**0.03***	0.00	0.07
	Sexuality (ref. HET)	-0.01	0.05	-0.26	0.79	-0.12	0.09
	Sex (ref. males)	0.01	0.03	0.19	0.85	-0.06	0.07
	Mig. background (ref. natives)	-0.02	0.03	-0.52	0.61	-0.08	0.04
	ISEI (ref. below median)	-0.01	0.03	-0.23	0.82	-0.07	0.06
	Education (ref. higher education)	0.02	0.03	0.69	0.49	-0.04	0.09
	Exposure	0.07	0.03	2.64	**0.01****	0.02	0.12
	Sensation seeking	0.02	0.01	1.22	0.22	-0.01	0.05
	Low self-control	0.02	0.01	1.32	0.19	-0.01	0.05
	Inter. Symptoms	0.10	0.01	7.14	**<.001*****	0.08	0.13
	Bullying	0.01	0.01	0.74	0.46	-0.02	0.04
	Leisure	0.00	0.01	-0.04	0.97	-0.03	0.03
	Sexuality*Sex	0.02	0.07	0.29	0.77	-0.11	0.15
Opioids	(Intercept)	1.27	0.06	22.73	**<.001*****	1.16	1.38
	Age	0.05	0.02	2.12	**0.03***	0.00	0.09
	Sexuality (ref. HET)	-0.13	0.07	-1.83	0.07	-0.27	0.01
	Sex (ref. males)	0.02	0.04	0.42	0.67	-0.07	0.10
	Mig. background (ref. natives)	-0.02	0.04	-0.41	0.68	-0.10	0.06
	ISEI (ref. below median)	-0.05	0.04	-1.27	0.20	-0.14	0.03
	Education (ref. higher education)	0.06	0.04	1.42	0.16	-0.02	0.14
	Exposure	0.01	0.04	0.36	0.72	-0.06	0.08
	Sensation seeking	0.00	0.02	0.25	0.80	-0.03	0.04
	Low self-control	0.07	0.02	3.90	**<.001*****	0.04	0.11
	Inter. Symptoms	0.11	0.02	5.52	**<.001*****	0.07	0.14
	Bullying	0.03	0.02	1.77	0.08	0.00	0.07
	Leisure	0.01	0.02	0.45	0.66	-0.03	0.04
	Sexuality*Sex	0.05	0.09	0.58	0.56	-0.12	0.22
**Logistic Regressions (Polysubstance use)**							
**Quadratic Regressions**							
Poly 1	(Intercept)	1.02	0.33	3.06	**0.00****	0.37	1.68
	Age	0.09	0.07	1.21	0.23	-0.05	0.23
	Age^2	-0.24	0.10	-2.39	**0.02***	-0.44	-0.04
	Sexuality (ref. HET)	-0.32	0.41	-0.77	0.44	-1.12	0.49
	Sex (ref. males)	-0.22	0.24	-0.90	0.37	-0.69	0.26
	Mig. background (ref. natives)	-0.09	0.24	-0.39	0.70	-0.57	0.38
	ISEI (ref. below median)	0.24	0.25	0.95	0.34	-0.25	0.73

Outcome	Predictor	Estimate	Standard Error	t-value	p-value	CI LL	CI UL
	Education (ref. higher education)	0.01	0.24	0.04	0.97	-0.47	0.48
	Exposure	2.42	0.19	12.83	**<.001*** **	2.05	2.78
	Sensation seeking	0.24	0.11	2.13	**0.03***	0.02	0.46
	Low self-control	0.52	0.10	5.18	**<.001*** **	0.32	0.71
	Inter. Symptoms	0.08	0.10	0.84	0.40	-0.11	0.28
	Bullying	0.10	0.09	1.04	0.30	-0.08	0.27
	Leisure	0.54	0.09	5.89	**<.001*** **	0.36	0.72
	Sexuality*Sex	1.09	0.50	2.17	**0.03***	0.11	2.08
Poly 2	(Intercept)	-3.90	0.39	-9.96	**<.001*** **	-4.67	-3.13
	Age	0.97	0.11	9.21	**<.001*** **	0.76	1.17
	Age^2	-0.57	0.12	-4.93	**<.001*** **	-0.80	-0.34
	Sexuality (ref. HET)	0.20	0.35	0.58	0.56	-0.48	0.88
	Sex (ref. males)	-1.14	0.27	-4.24	**<.001*** **	-1.67	-0.61
	Mig. background (ref. natives)	-0.80	0.24	-3.27	**0.00****	-1.27	-0.32
	ISEI (ref. below median)	0.23	0.25	0.91	0.36	-0.26	0.71
	Education (ref. higher education)	0.35	0.26	1.36	0.17	-0.15	0.85
	Exposure	2.41	0.26	9.19	**<.001*** **	1.90	2.93
	Sensation seeking	0.32	0.11	2.78	**0.01****	0.09	0.54
	Low self-control	0.61	0.10	5.96	**<.001*** **	0.41	0.81
	Inter. Symptoms	0.27	0.10	2.63	**0.01****	0.07	0.47
	Bullying	0.01	0.09	0.06	0.95	-0.18	0.19
	Leisure	0.57	0.10	5.55	**<.001*** **	0.37	0.77
	Sexuality*Sex	1.06	0.45	2.37	**0.02***	0.18	1.94
**Linear Regression**							
Poly3	(Intercept)	-1.75	0.28	-6.20	**<.001*** **	-2.30	-1.19
	Age	0.13	0.10	1.34	0.18	-0.06	0.33
	Sexuality (ref. HET)	0.03	0.32	0.08	0.93	-0.59	0.65
	Sex (ref. males)	-0.93	0.22	-4.28	**<.001*** **	-1.36	-0.51
	Mig. background (ref. natives)	-0.42	0.20	-2.12	**0.03***	-0.81	-0.03
	ISEI (ref. below median)	0.23	0.21	1.13	0.26	-0.17	0.64
	Education (ref. higher education)	0.10	0.21	0.49	0.63	-0.31	0.52
	Exposure	1.86	0.18	10.22	**<.001*** **	1.50	2.21
	Sensation seeking	0.23	0.09	2.41	**0.02***	0.04	0.41
	Low self-control	0.45	0.09	5.04	**<.001*** **	0.28	0.63
	Inter. Symptoms	0.44	0.09	4.69	**<.001*** **	0.26	0.62
	Bullying	0.06	0.08	0.69	0.49	-0.11	0.22
	Leisure	0.34	0.09	3.79	**<.001*** **	0.16	0.51
	Sexuality*Sex	0.84	0.40	2.11	**0.03***	0.06	1.62

Note: For substances available at only two time points (benzodiazepines, opioids, and poly 3), linear regression was used. For the other substances, quadratic regression models were applied.

Table S4c. Overview of significant predictors. Full models (baseline + demographics + control) with interaction terms.

**Substace /Predictor**	**Tobac-co**	**Alco-hol**	**Canna -bis**	**Ecsta-sy**	**Stimu-lants**	**Cocain e**	**(Meth-) Amphe - tamine s**	**Hallu-cinoge ns**	**Benzo-dize-pines**	**Opi-oids**	**Poly 1**	**Poly 2**	**Poly 3**	**Sum +**	**Sum -**
**Baseline**															
Age		+		+	+	+	+	+	+	+		+		9/13	0/13
Age^2		-		-	-	-	-	-	*NA*	*NA*	-	-	*NA*	0/10	8/10
Sexuality (ref. HET)				+	+		+	+						4/13	0/13
Sex (ref. males)		-	-		-	-						-	-	0/13	6/13
**Sociodemographics**															
Mig. background (ref. natives)	+	-		-	-	-	-	-				-	-	1/13	8/13
ISEI (ref. below median)		+	+											2/13	0/13
Education (ref. higher education)	+	-		+	+	+								3/13	1/13
**Psychosocial Predictors**															
Peer SU	+	+	+	+	+	+	+	+	+		+	+	+	12/13	0/13
Sensation seeking			+	+	+	+	+	+			+	+	+	9/13	0/13
Low self-control	+	+	+	+	+	+	+	+		+	+	+	+	12/13	0/13
Inter. Symptoms			+		+	+	+		+	+		+	+	7/13	0/13
Bullying	+				+	+	+							4/13	0/13
Leisure	+	+	+	+	+	+	+	+			+	+	+	11/13	0/13
Sexuality *Sex			+					-			+	+	+	4/13	1/13

Note: + indicating a positive association, - a negative association. For substances available at only two time points (benzodiazepines, opioids, and poly 3), linear regression was used. For the other substances, quadratic regression models were applied. ISEI = International Socio-Economic Index of Occupational Status.

Figure S1. Trajectories of Ecstasy, cocaine, (meth-)amphetamine, stimulant, hallucinogen, opioid, benzodiazepine, and poly2 use with no significant difference between heterosexual (HET) and sexual minority youth and young adults (SM) from ages 17 to 24.

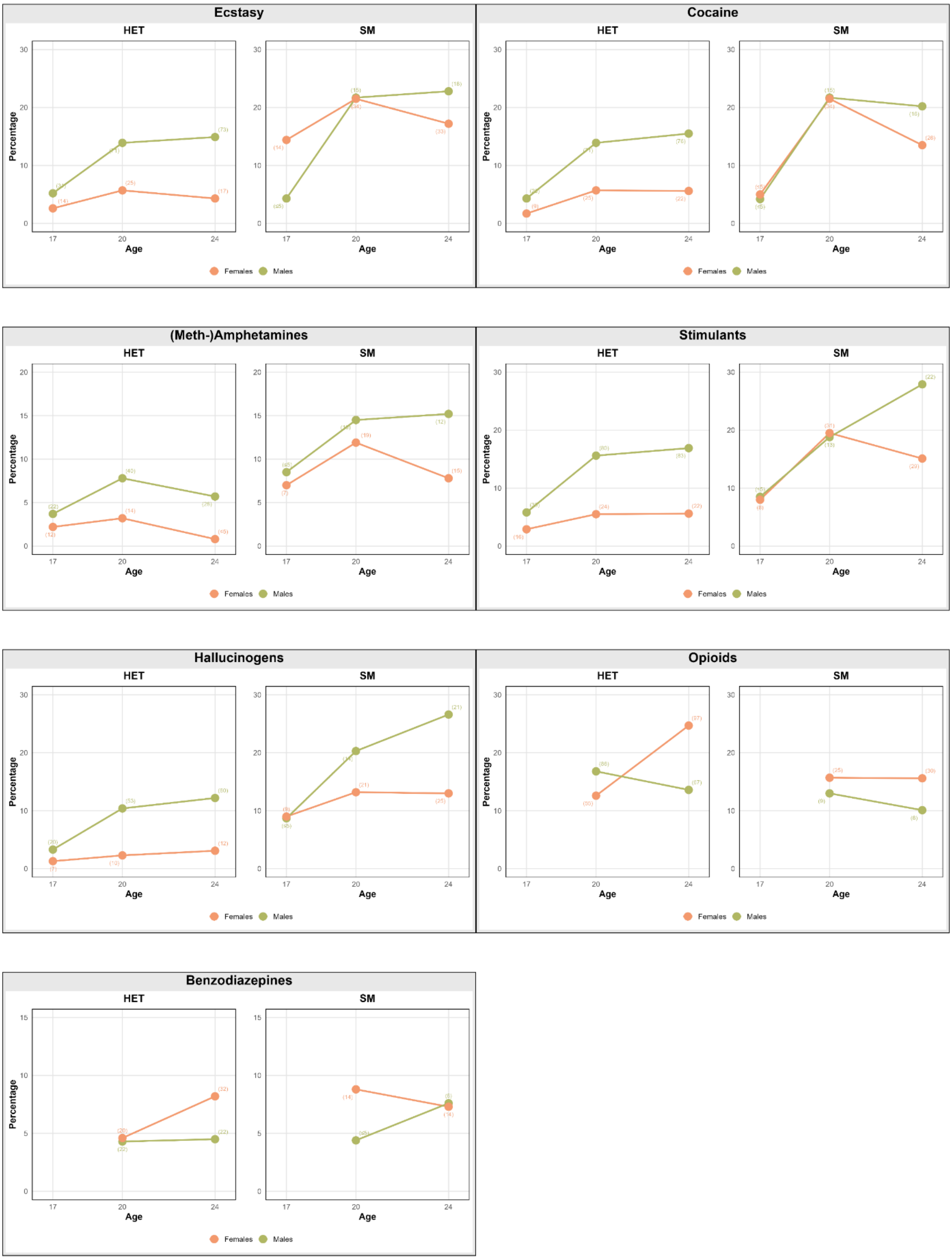

Figure S2a. Trajectories of tobacco, alcohol, and cannabis use among male and female youth and young adults, stratified by sexual attraction (HET vs. SM) from ages 17 to 24.

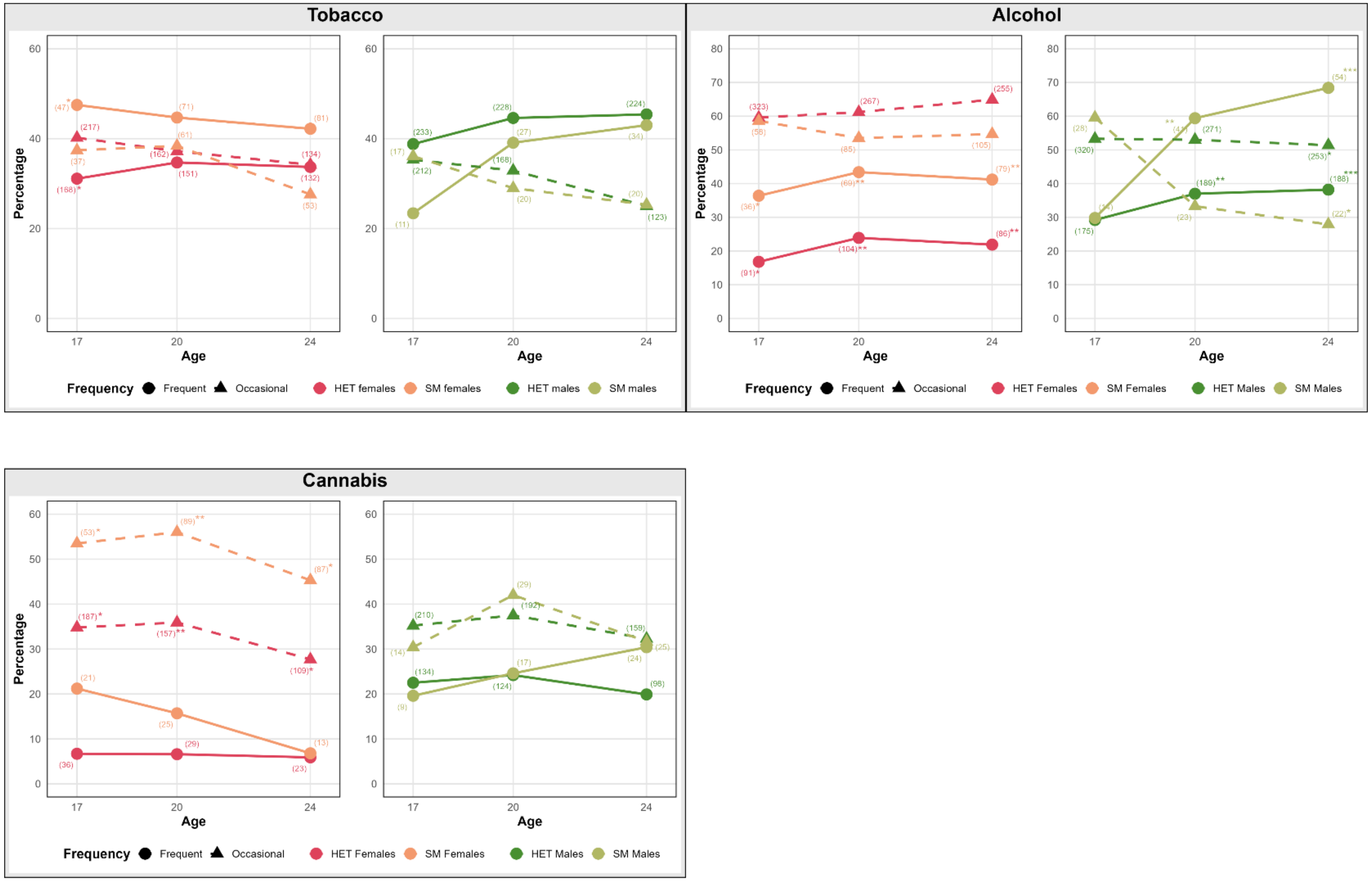

Note: Asterisks indicate significance. *= p <.05, **= p <.01, *** = p <.001.

Figure S2b. Trajectories of Ecstasy, cocaine, (meth-)amphetamine, stimulant, hallucinogen, opioid, and benzodiazepine use among male and female youth and young adults, stratified by sexual attraction (HET vs. SM) from ages 17 to 24.

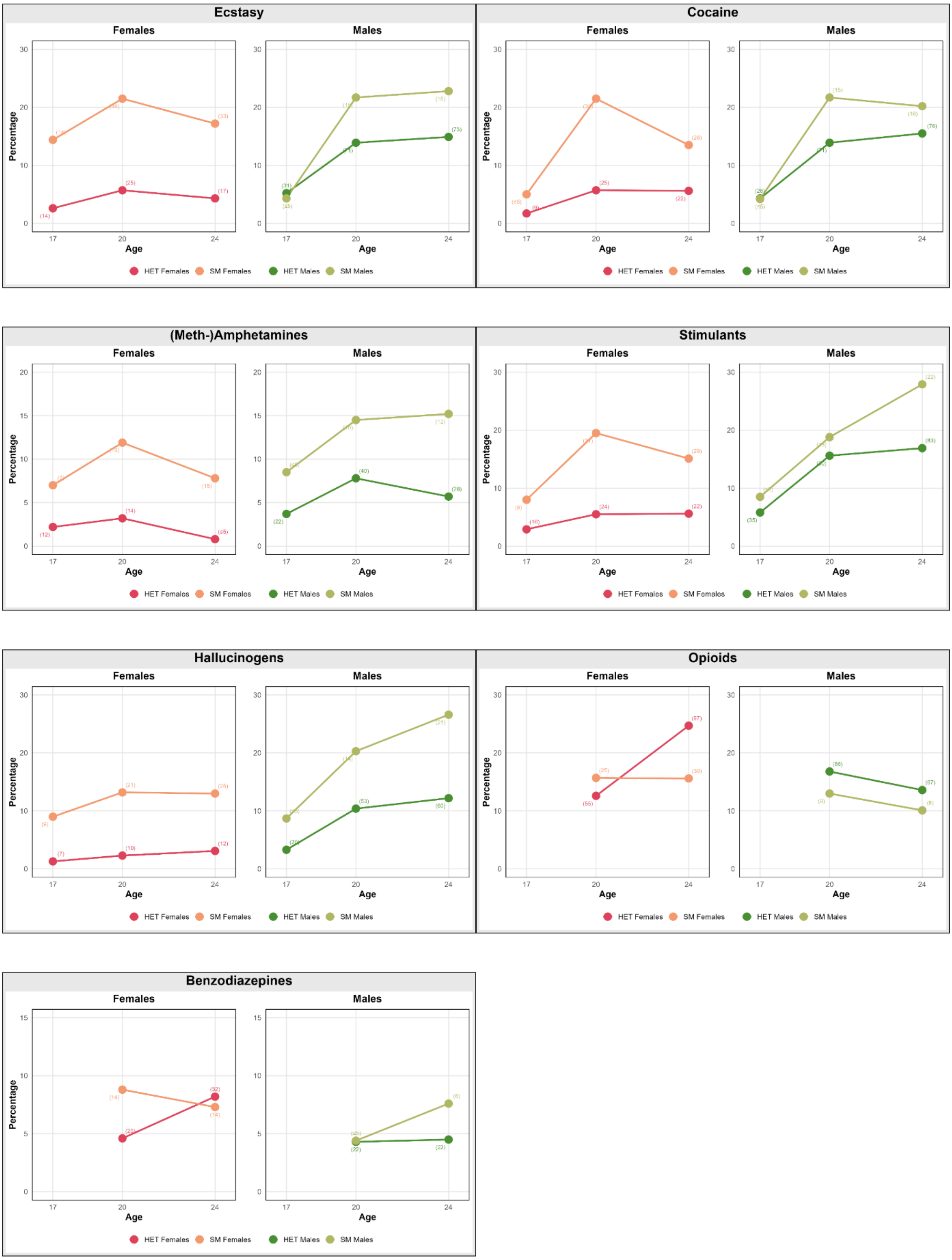

Note: Asterisks indicate significance. *= p <.05, **= p <.01, *** = p <.001.

Figure S2c. Trajectories of polysubstance use (Poly1-3) among male and female youth and young adults, stratified by sexual attraction (HET vs. SM) from ages 17 to 24.

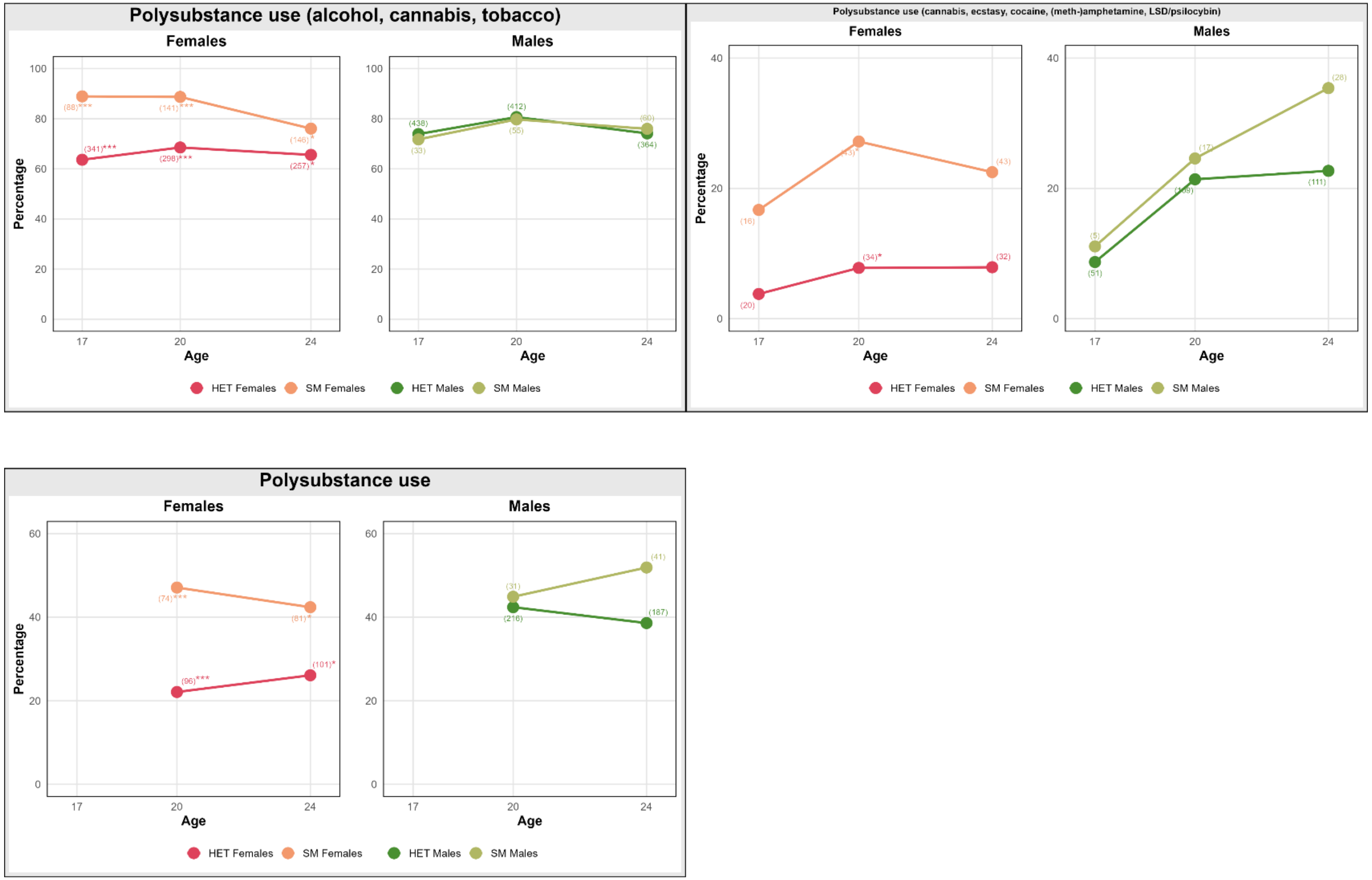

Note: Asterisks indicate significance. *= p <.05, **= p <.01, *** = p <.001
